# Faculty-Led Study Abroad Programs and Learning Sustainability: A Southeast Asian Context

**DOI:** 10.12688/f1000research.178945.1

**Published:** 2026-04-10

**Authors:** Bama Andika Putra

**Affiliations:** 1 University of Bristol School of Sociology Politics and International Studies, Bristol, England, UK; 2 Universitas Hasanuddin Fakultas Ilmu Sosial dan Ilmu Politik, Makassar, South Sulawesi, Indonesia

**Keywords:** Study Abroad, Sustainability, Southeast Asia, International Relations, Pedagogy, Experiential Learning

## Abstract

As a widely offered course in International Relations (IR) programs, the teaching and learning of Southeast Asian Regional Dynamics (SARD) struggles to adopt a pedagogical strategy that counters stagnancy and passive learning. The problem is exacerbated when SARD courses discuss topics of sustainability in Southeast Asia, which obligates instructors to introduce complex empirical cases that may be difficult for students to grasp. As a means to ignite the students’ interests and allow a platform in which students can see directly the issues that defy sustainable development in the region, this study bridges the conception of experiential learning in SARD courses based on Below, Nydegger, and Parmentier’s study on short-term faculty-led study abroad programs, and looks at the prospects of its implementation into SARD courses. To make this argument, this study explores the following ideas: The intertwining of sustainability and Southeast Asian contexts by exploring academic topics such as haze pollution, climate change, and illegal fishing within the context of Southeast Asia, as well as culturally-sensitive topics; acknowledgement of the instructors’ responsibilities within the context of program planning, technical arrangements, and the conduct of program orientations; and establishing active learning students through real-life learning mechanisms that elevate the students’ independent learning, research skills, communication skills, and critical thinking.

## 1. Introduction

Within the context of IR teaching and learning programs of higher education institutions, sustainability-related topics have always been an integral part of the curricula. In some universities, sustainability acts as a single elective course; meanwhile, in others, it is part of a broader IR topic. For example, in a course discussing regional dynamics of IR, a course on SARD may have a topic discussing how sustainability has been achieved with regional collaborative efforts within the Association of Southeast Asian Nations (ASEAN) and the tensions arising among the states within that region.

Nevertheless, the idea of sustainability differs from other analytical and conceptual frameworks within the study of IR. The United Nations Educational, Scientific, and Cultural Organization (UNESCO) announced the framework of Education for Sustainable Development as a global development agenda of ingraining sustainable development into the curricula of higher education institutions ( Glavič, 2020; Mochizuki & Vickers, 2024; Oe et al., 2022; UNESCO, 2024; Velempini & Maruatona, 2025). Consequently, UNESCO expects students to be able to develop the relevant knowledge and skills in relation to sustainable development, considering the high probability of the globe’s sustainability measures being in the students’ hands in a couple of years ( Elias & Bangkok, 2006; Glavič, 2020; Kohl et al., 2022). Therefore, in higher education institutions globally, there is a growing concern about how to best teach sustainability in a way that enables the enhancement of students’ behaviors and skills in alignment with sustainable development.

However, in the case of IR teaching and learning, an issue is immediately encountered. IR classrooms, including those that cover sustainability topics, tend to focus on theoretical, conceptual, and empirical issues through traditional teaching methods in classrooms ( Bernstein, 2012; Lüdert, 2016; Campillo, Hernandez, and Cantano, 2021; Putra, 2025). Without denying the benefits of traditional teaching methods, the case of learning sustainability presents its distinct challenges. Students need to see directly with their own eyes issues that defy sustainable development to develop the behaviors and knowledge on the topic truly. As argued by many scholars in the past, this has been the reason why many universities globally adopt service-learning as a form of experiential learning, where there is a combination of both in-classroom teaching and community service ( Álvarez-Vanegas et al., 2024; Aramburuzabala, 2025; Aramburuzabala & Cerrillo, 2023; Ribeiro et al., 2023). Unfortunately, such a theme remains one that is unexplored within IR academia, considering that the focus on IR pedagogy as of late has been confined to student-centered learning approaches such as simulations, debates, discussions, and posters ( Duchin & Sherwood, 1990; Newmann & Twigg, 2000; Sasley, 2010; Sousa & Clark, 2019; Taylor, 2013; Wheeler, 2006).

The case is also evident in the teaching and learning processes of SARD courses. As a widely taught course in many Southeast Asian universities, SARD also struggles with the adoption of alternative pedagogies to replace stagnant and passive learning that tends to be adopted in classrooms. Therefore, although students attain greater knowledge of the sustainability concerns in the Southeast Asian region by the end of the course, the course is unable to offer direct experiences with global sustainability issues, as its focus tends to be oriented toward empirical and theoretical explanations. This is a concern, considering the trend of higher education institutions undertaking systematic changes to orient research and education practices to sustainability ( Chou & Vun, 2025; Mensah, 2019; Wals, 2014). As a study in 2021 argued, “[…] universities as priority organisations and agents of change within the sphere of their social commitment” ( Prieto-Jiménez et al., 2021, p. 1).

Drawing on relevant literature on experiential learning and faculty-led study abroad programs, this study argues for the relevance of short-term study abroad programs as a means to supplement what is taught within SARD classes. Considering the complex nature of the topic, which comprises dynamic tensions among Southeast Asian states due to haze pollution, the difficulty of balancing economic performances and environmental protection, as well as challenges of climate change, this study perceives that students could benefit from a short-term abroad program that helps them work on a specific case related to sustainability. As argued by many scholars in the past, experiential learning provides a unique learning atmosphere for students as they delve at the center of the community to observe by themselves, dynamics related to the substantives being taught in classrooms ( Below et al., 2021; Blewitt et al., 2021; Kolb, 1984; Pipitone, 2018; Strange & Gibson, 2017). As a means to bridge the conception of experiential learning in the SARD courses, this study is informed by Amy Below, Amanda Nydegger, and Mary Jane Parmentier’s ‘Experiential Learning through Faculty-led Study Abroad Programs’ and looks to implement the program conceptions of the study into SARD courses.

Experiential learning in IR is not new. However, it is less common within the specific context of SARD. In the past, scholars have argued that service learning offered as an experiential learning experience has been able to ignite the interests of students towards sustainable development in alignment with UNESCO’s Education for Sustainable Development ( Elias & Bangkok, 2006; Mochizuki & Vickers, 2024; Oe et al., 2022; UNESCO, 2024). This study aims to build on that by arguing how SARD courses can benefit from a short-term abroad program for those selecting the course, as a means to broaden the students’ horizon and look at the issues related to the nexus between sustainability and the Southeast Asian region beyond the lens of issues taking place within their country.

## 2. Learning sustainability: A literature review

Learning sustainability is not a simple task for the typical student. As Kleespies and Dierkes found in their 2022 study, a connection exists between social, economic, and environmental factors in terms of sustainability ( Kleespies & Dierkes, 2022), which leads to a multitude of issues affecting various actors. Therefore, in assessing alternative pedagogy in SARD courses, this study perceives the following discourses as relevant to this study. They include the discourse on the barriers to learning sustainability, divergent methods adopted by instructors in the past, as well as the unique benefits of using service-learning within the context of understanding sustainable development. Combined, the argument presented in this section is that existing scholarship has not made the applicability of experiential learning methods available in region-specific cases, such as the SARD course, and seeks to adopt experiential methods to enhance students’ experiences.

The first discourse relevant to this study examines the numerous barriers to learning sustainability. Within this discourse, scholars acknowledge that sustainability is a complex topic, which leads to different variables affecting the success of the transfer of knowledge intended by instructors ( Grund et al., 2023; Parry & Metzger, 2023; Silva-Jean & Kneippb, 2024; Stam et al., 2023). As Stam, Ewjik, and Chan found in 2023, there is a complex relationship in the topic of ‘sustainability transitions,’ and a review is needed to assess whether the learning processes have led to more sustainable behaviors among students ( Stam, van Ewijk & Chan, 2023, p. 1). In a more general context, Perry and Metzger argued that the barriers to sustainable learning can be defined as comprising multiple variables, such as “[…] disciplinary silos, […] high stakes assessments, and inadequate professional learning opportunities” ( Parry & Metzger, 2023, p. 1). The study is unique, as it centers on the concerns of the instructors’ lack of preparation and competencies in relation to the teaching and learning processes of sustainable development.

Nevertheless, there is also considerable work examining other variables such as emotions, social learning, agency, and curriculum as the main barriers to sustainability learning ( Araneo, 2024; Grund et al., 2023; Koskela & Paloniemi, 2023; Silva-Jean & Kneippb, 2024). On the variable of emotions, for example, a 2023 study called for an “[…] educational practice where emotions can be experienced, expressed, and understood in a safe atmosphere” ( Grund, Singer-Brodowski and Büssing, 2023, p. 307), after arguing that emotions affect transformative learning processes. Looking at the issues of sustainability that students found interesting, Araneo’s study found that sustainability curricula can be tweaked to ensure an alignment with students’ interests based on the general categories of sustainability-based sciences, relevant contexts, and the hope for the future ( Araneo, 2024). Meanwhile, looking at the issue of sustainability learning from the lens of human agency theory, a study in 2023 argued the importance on reflecting on the concept of agency, and how such reflections can lead to “[…] countless actions in individual lives, communities, and local, national and global scenes can contribute to sustainability” ( Koskela & Paloniemi, 2023, p. 164).

Despite the rich theoretical and academic discussions on the barriers of sustainability learning, there have also been considerable studies made exploring divergent methods. Scholars have explored different approaches in teaching and learning sustainability, including community-based programs, problem-based learning, collaborative mechanisms, innovative pedagogies, as well as virtual experiences ( Bremner & Steed, 2025; Carrió Llach & Llerena Bastida, 2023; Gesthuizen et al., 2025; Nguyen et al., 2024; Shah et al., 2024). For example, studies on problem-based learning show that students are able to express more of their emotions due to the connection between the problem and real-life events ( Carrió Llach & Llerena Bastida, 2023; Nguyen et al., 2024).

In other cases, academics have explored various methods to establish practical approaches in teaching and learning sustainability. An example, through collaborative mechanisms with relevant institutions, a 2025 study concluded that knowledge on Sustainable Development Goals (SDGs) can be retrieved by bringing, “[ …] students from other cultures and slightly different disciplines, with one being management and the other being practice-based, together to successfully demonstrate the use of an innovative methods” ( Bremner & Steed, 2025, p. 171). Meanwhile, other studies, such as Howell, introduced the use of the ‘flipped classroom’ as a means to establish “[…] reflective and active learning” ( Howell, 2021, p. 1). In a more contemporary setting and incorporating modern technology, Gesthuizen, Tan, and Kidman found the effective use of virtual platforms in designing sustainable virtual buildings and landscapes, as a means to establish “[…] future-thinking mindsets” ( Gesthuizen, Tan, and Kidman, 2025, p. 25).

Nevertheless, the majority of studies have focused on the more traditional methods of teaching and learning processes that help establish emotional connections with students’ surroundings. As mentioned in multiple studies looking at community service programs being implemented in higher education institutions, these studies are united by the argument that these methods allow students to actively engage with their local communities and help in “[…] fostering civic responsibility” ( Shah et al., 2024, p. 1).

The last discourse on service-learning encompasses the majority of studies examining sustainability learning methods. Through a service-learning pedagogical approach, students can enhance their knowledge of the SDGs and practices that promote sustainable community development ( Brower, 2011; Ribeiro et al., 2023). Indeed, numerous studies in the past have established strong connections between service-learning and the eventual development of students’ awareness regarding the elements and dynamics of sustainability ( Pearce and Manion, 2016; Aramburuzabala and Cerrillo, 2023; Álvarez-Vanegas, Ramani, and Volante, 2024; Antal, 2024; Rodríguez-Zurita et al., 2025).

Specific studies would only differ in the points being investigated in relation to service-learning. In a 2024 study, academics traced the processes leading to the substantial shift in curriculum pertaining to sustainable development studies, which have now introduced more experiential learning methods ( Rodríguez-Zurita et al., 2025, p. 158). Meanwhile, a 2016 study highlighted the benefits of service-learning, using the example of community-based service-learning projects conducted at Virginia Tech ( Pearce & Manion, 2016). Service-learning, therefore, has established itself as one of the signature pedagogies for disseminating knowledge in sustainability studies, which many scholars have trusted in the past to raise students’ awareness ( Álvarez-Vanegas, Ramani, and Volante, 2024; Aramburuzabala, 2025; Narong, 2025).

But how about the teaching and learning of sustainability in the context of a different curriculum, specifically SARD courses in IR? Unfortunately, the processes are not straightforward, as there has been no academic inquiry specifically addressing the various contexts and challenges associated with increasing the awareness of sustainability topics and concerns among IR students in the Southeast Asian regional context. In a study made in 2024, scholars acknowledged the importance of educators as the leading actor that helps to determine a successful learning system for students, stating, “Educators play a pivotal role in implementation, and unless they are trained and incentivized and this is systemized, not only Service-Learning but also ESD may fail to transform learning environments” ( Álvarez-Vanegas, Ramani & Volante, 2024, p. 1). Similarly, Perry and Metzger argued, “[…] research shows that many practicing educators feel unprepared to help learners develop the competencies needed to forge more sustainable paths forward” ( Parry & Metzger, 2023, p. 1). Consequently, although this study recognizes the positive impact of service-learning within the context of learning sustainability, its implementation within SARD courses under IR programs is not a straightforward process. Educators should therefore be provided with guidelines on how to adopt such methods within specific contexts.

## 3. Enhancing Southeast Asian regional dynamics and sustainable development learning: Adopting experiential learning

Recognizing the lack of past studies that cover the ‘how’ of learning sustainability within the context of SARD courses under IR programs, this study bridges the conception of experiential learning through short-term abroad programs, as proposed by Below, Nydegger, and Parmentier. Within the discourse of experiential learning, numerous studies in the past have argued how study abroad programs allows for greater achievement of a subject’s learning outcomes and advance students’ cross-cultural awareness ( Anderson et al., 2019; Bai et al., 2016; Edmunds & Shore, 2020; Giedt et al., 2015; Mule et al., 2018; Strange & Gibson, 2017; M. Tarrant & Lyons, 2012). As some scholars have noted, however, these faculty-led programs must be carefully formulated by instructors to achieve the expected outcomes, as positive outcomes are not automatic ( Parmentier and Moore, 2016; Mule, Audley, and Aloisio, 2018; Below, Nydegger, and Parmentier, 2021). Therefore, as this section aims to provide such a guideline, the ideas are inspired by the experiences of Below, Nydegger, and Parmentier in running short-term study abroad programs in Morocco and the Dominican Republic.

The first looks to construct the outline of a program, which comprises the program design and curriculum. Perhaps the most significant task is to determine the destination and length of a short-term abroad program as an experiential learning experience for students ( Below et al., 2021). Some questions to consider before announcing the program include whether students have access to scholarships from within or outside the university, and in which term the program would be best offered to students. Regarding curriculum, the substantive focus would vary significantly between experiential learning programs. This should be based on the learning outcomes of a given course, to ensure alignment between what is being taught in the classroom and the actual program hosted abroad by the university. Nevertheless, as scholars have pointed out, there should be a balanced blend between subject-focused discussions and cross-cultural and interdisciplinary approaches in the program ( Tarrant, 2010; Tarrant and Lyons, 2012; Bell et al., 2016; Anderson, Dore-Welch, and Johnson, 2019; Below, Nydegger, and Parmentier, 2021). Other considerations should include the coverage of the discussions and background of the students ( Below et al., 2021; Mitchell & Maloff, 2016; Malewski & Phillion, 2009; Pipitone, 2018).

Besides determining the substantive coverage of experiential learning, instructors should also be aware of other responsibilities. From the onset, instructors should be able to answer several technical questions before initiating the program. In relation to the institution, it is essential to determine whether there are significant barriers and challenges within the university or department ( Howard and Keller, 2009; Below, Nydegger, and Parmentier, 2021). Once a program has taken shape, it is essential for the instructors also to determine whether there are potential partnering institutions abroad that could cooperate in the conduct of this short-term study abroad program ( Below, Nydegger, and Parmentier, 2021). One solution could be to ask the university’s partnerships department to compile a list of universities that are interested in cooperation, and then shortlist the potential destinations after liaising with those institutions to determine their suitability for hosting students.

Another responsibility for the instructors pertains to the technical elements of the travel. Regarding factors related to accommodation, in-city transportation, and airport pickup, these technical aspects could be overwhelming for the instructors. Therefore, as suggested in several past studies, instructors could benefit from using a third-party service or a ‘provider’ that can help arrange the technical elements in a host country where an instructor may not be very familiar ( Eckert et al., 2016; Below, Nydegger, and Parmentier, 2021). A distinct benefit of using providers is that they can provide emergency support for the delegation, and help the instructors by being the actors that “[…] knows the language, understands the legal systems, and can garner additional support and resources in the case of an emergency” ( Below, Nydegger, and Parmentier, 2021, p. 82).

Instructors are also placed with the burden of having multiple roles in the program. Not only is the expectation that instructors will lead the effort in determining the program outline, but they are also expected to act as a health professional for the students in times of emergency ( Below, Nydegger, and Parmentier, 2021). Instructors should therefore prepare themselves to undertake roles to respond to issues related to mental health, considering how the students would be exposed to new environments and can easily encounter cultural shocks ( Alkubaidi & Alzhrani, 2020; Demes & Geeraert, 2015; EL-ASRI et al., 2024; Tekel et al., 2025). One way to mitigate this, as recommended by Below, Nydegger, and Parmentier, is through the conduct of pre- and re-entry orientations. In both orientations, instructors have the opportunity to provide basic knowledge on what to expect throughout the program (subject and cultural-related), as well as prepare the students to return to their everyday lives after the completion of the program ( Bai et al., 2016; Below et al., 2021; C. J. L. Campbell et al., 2015; Giedt et al., 2015; Paras et al., 2019).

The pedagogical strategy used throughout the short-term program would also need to be carefully determined by the instructors. As argued in the literature review section, multiple studies have supported the positive impacts of using service-learning as a means to enhance students’ learning experiences in the past ( Aramburuzabala and Cerrillo, 2023; Álvarez-Vanegas, Ramani, and Volante, 2024; Aramburuzabala, 2025). Would there be a traditional in-class teaching experience offered in the program? Would the assignments be individual or group-based? What assignment activities would be given to the students? ( Below et al., 2021; Gough et al., 2018). These are all technical elements of the short-term abroad program that instructors need to be aware of.

Finally, several key points regarding the responsibilities of students need to be highlighted. Before their departure, one significant barrier that students will need to overcome is the costs associated with the program. As a means to enhance real-life skills ( Granato et al., 2024; Lien, 2007; Lien & Liu, 2010; Oduwaye et al., 2023), students would need to prepare their own funding to attend the program if a scholarship is not available ( Charlotte, 2019). Consequently, they will need to enhance their creative thinking by spreading out proposals or making joint fundraising programs to ensure that the cost barrier can be overcome.

Another issue that students need to be aware of is to prepare themselves with the relevant materials related to the program. In the pre-departure program, students must ensure that they properly engage with the subject matter and culturally related topics covered to ensure they are prepared to participate in learning activities abroad. Meanwhile, throughout the program’s conduct, students are expected to be at the center of the learning process and display active participation when hosted by another university ( Below, Nydegger, and Parmentier, 2021). Consequently, it is expected that students will remain active throughout the study abroad program and display respect for the unique cultural customs adopted in the foreign country.

## 4. Faculty-led study abroad programs: Alternative methods in learning sustainability in the context of Southeast Asian regional dynamics

The ideas introduced by Below, Nydegger, and Parmentier will be integrated into the teaching and learning process of SARD courses within IR programs. In doing so, the following will be structured as follows. First, it provides a recommendation for a program’s outline, aiming to intertwine the fundamentals of sustainability with Southeast Asian contexts for an abroad program. Following this is an elaboration of the instructors’ responsibilities and what is expected from a participating student. By providing such guidelines, this study benefits instructors who seek to adopt experiential learning and integrate creative pedagogies with service-learning in their teaching and learning practices.

### 4.1. Outline of a Program: Ideas for intertwining sustainability and Southeast Asian contexts

The substantive coverage of an SARD course encompasses a wide range of empirical issues within the Southeast Asian region. A typical SARD course would begin by covering the history of the region, the vast political dynamics of the member states, efforts toward regional integration, as well as the contemporary challenges faced by the Southeast Asian states. In relation to sustainability, therefore, SARD courses would only cover the topic as a minor class session or across several classes as an example of cooperation or as an illustration of barriers to regional engagement.

Consequently, it is essential to identify several potential topics related to sustainability within the Southeast Asian context, which will serve as the basis for teaching and learning subjects offered in the short-term study abroad program. One of the first issues that instructors and students can explore is haze pollution in Southeast Asia. The haze problem is unique in the region, as it is a complex issue involving the roles of local, state, and regional actors ( Gan et al., 2021; Wang et al., 2023; Wangwongwatana, 2023; Y. Zhao et al., 2023). Indonesia, being one of the primary sources of haze in the region, has maintained the position that the sources of haze pollution also originate from other Southeast Asian states, which makes an Indonesian-specific solution unfair ( ASEAN, 2024a; Putra, 2024b; Varkkey, 2024). Regional organizations, such as ASEAN, have implemented numerous policies in the past to foster cooperation among Southeast Asian states, considering the significant environmental and health impacts that haze pollution causes ( ASEAN, 2024a, 2024b, 2024c; Varkkey, 2024). Students could explore potential solutions to haze pollution, taking into consideration the complexities of negotiations on the resolution over the past two decades.

Another topic that students can explore in the teaching and learning platforms of a study abroad program related to SARD is the issue of illegal, unreported, and unregulated fishing (IUUF). The Southeast Asian region is rich in sea-based resources, which the states in the region continue to exploit ( Fleck, 2024; Parameswaran, 2017; Resosudarmo & Kosadi, 2018; Schlieman, 2023). However, in recent years, there has been a considerable issue of rising IUUF in Southeast Asian waters, which severely undermines the Southeast Asian states’ fisheries resources ( Chapsos et al., 2019; InvestSEA, 2023; IOJI, 2022; Noviansyah, 2018; Schlieman, 2023; Xiang, 2018). Besides the difficulty of counter IUUF in Southeast Asia, there is also the issue of overlapping Exclusive Economic Zones among states in the region, which has led to multiple episodes of fishing boats infiltrating the seas of other states to conduct fisheries activities ( BBC, 2016; Bhwana, 2020; IOJI, 2022; Juwana, 2014; Vu, 2020). Therefore, this environmental issue has a complex political context that necessitates thorough exploration to fully understand its nature and potential solutions.

Another topic that intertwines the issues of sustainability and the Southeast Asian regional context is climate change. Climate change is a global issue, not confined to any particular region. However, the dynamics in Southeast Asia are unique. First, Southeast Asian states are currently at a phase where industrialization is a trend among the government stakeholders, as they undertake measures to enhance the state’s economy by establishing industrialized systems ( de Pleijt & Frankema, 2025; Huq & Ichihashi, 2025; Kikuchi, 2018; Rasiah & Yun, 2009; X. Zhao, 2025). Consequently, this has led to practices that can be considered non-environmentally friendly, as state and private actors aim to achieve a particular target with little regard for the environment ( Rasiah & Yun, 2009; Zafar et al., 2020; Zafarullah & Mehnaz, 2025).

Within the context of Southeast Asia’s concerns over climate change are also the vast impacts that the region encounters. Southeast Asia primarily comprises littoral states and has direct boundaries with international seas. Consequently, in recent years, there has been a steady rise in sea levels directly linked to the global temperature increase ( IPCC, 2014; Lindsey, 2021). Another issue has been the increasing frequency of unpredictable weather in Southeast Asia, which has severely disrupted economic activities within the region’s states ( ADB, 2015; Prakash, 2018; Putra, 2024a; Suwandaru et al., 2024). Unfortunately, for typical IR students, it is challenging to grasp the impacts of climate change through a brief lecture in class. Through experiential learning, instructors can focus on the implications of climate change, allowing students to experience the issue firsthand, whether through direct observation or by exploring society and discussing how the issue affects local communities.

As mentioned in multiple studies in the past ( Anderson et al., 2019; Below et al., 2021), there is also a need for the substances offered to be balanced with the topic of cultural awareness. Considering that students in a short-term study abroad program will visit a foreign country, considerable time should be invested in creating a curriculum that encompasses the diversity of the host country’s culture and prepares students for what they will encounter. This includes issues that may be culturally sensitive, as well as the use of specific terminologies that should be avoided ( Bobel, Al Hinai, and Roslani, 2022; Turkson-Ocran et al., 2022; Narayan et al., 2023). Within the context of SARD and sustainability, there are also similar concerns that warrant consideration. Local communities may be sensitive to some of the problems being discussed in the context of sustainability, which relate to issues such as climate change, IUUF, or haze pollution. Therefore, in addition to the basic cultural customs of a country, a program should also be aware of the discourses relevant to the case studies that will be assessed throughout the experiential learning experience.

### 4.2. Instructors’ responsibilities

After looking at the potential themes for the study abroad program, instructors would need to undertake several responsibilities to ensure the success of the experiential learning program. Before the actual departure of the students, instructors must first determine the timing of the program ( Madden, McMillan, and Madden, 2019; Below, Nydegger, and Parmentier, 2021). This includes the duration of the program and the month in which it will be offered to students. As mentioned by Charlotte West, considering the bearing costs, a short period (one or two weeks) should be sufficient to achieve the learning outcomes ( Charlotte, 2019). Meanwhile, the time offered within the academic year should be at the end of the SARD course. This allows students to gain a general and basic understanding of the regional dynamics within the region, and helps them analyze cases presented during the experiential learning.

As the responsible individual for the program, instructors would then need to look at the potential partnering universities to conduct the short-term study abroad program. Instructors should acknowledge that their universities have established partnerships with other higher education institutions in the past, with some of these partnerships remaining active in the current status quo. Therefore, instructors should be able to identify the options of host universities, taking into consideration that a particular higher education institution may have expertise and specialize in one of the themes that the instructors wish to emphasize in the study abroad program. Nevertheless, an instructor should be able to weigh in on different considerations in determining the host country and university. For example, in a SARD course, instructors should opt to look first at Southeast Asian Universities as the primary option, if it is technically possible to make that long journey. As SARD courses are mainly offered in Asian higher education institutions, the bearing costs are not expected to be excessive.

Meanwhile, during the conduct of the study abroad program, instructors are also faced with responsibilities related to the technical arrangements of the program and the pedagogical strategy used. On the former, the primary concern should be how instructors would arrange issues such as accommodations and transportation throughout the program. If the burden of this is unbearable, perhaps due to the high number of program participants, instructors should definitely explore the option of using providers ( Eckert et al., 2016; Below, Nydegger, and Parmentier, 2021). Despite the additional costs that must be paid, at least instructors can partially release themselves from the burden of technically arranging the trip once the delegations have arrived in the host country.

In doing so, a stronger emphasis can be placed on determining the most effective pedagogical strategy for the program. As mentioned in past studies, Below, Nydegger, and Parmentier stated, “[…] it is important to ensure our curricula are purposefully and holistically designed in order to have the positive impact we desire” ( Below, Nydegger, and Parmentier, 2021, p. 83). Instructors should then place a stronger emphasis on determining how the learning outcomes would be achieved by the conduct of the short-term study abroad program. To achieve this, it is essential to strike a balance between cultural substantives and interdisciplinary approaches in order to attain the desired outcomes ( Tarrant and Lyons, 2012; Bell et al., 2016; Anderson, Dore-Welch, and Johnson, 2019; Below, Nydegger, and Parmentier, 2021).

If instructors are looking to adopt a more interdisciplinary approach to their studies, perhaps examining innovative learning systems can provide the solution needed. As Below’s study argued, “[…] interdisciplinary approaches can facilitate rich conversations and learning experiences and attract students” ( Below, Nydegger and Parmentier, 2021, p. 83). Therefore, it would be beneficial to refer to past studies on alternative methods in teaching and learning sustainability, such as through problem-based learning, community-based learning, or quizzes ( Below et al., 2021; Bremner & Steed, 2025; Carrió Llach & Llerena Bastida, 2023; Howell, 2021; Nguyen et al., 2024; Shah et al., 2024). Meanwhile, instructors could also explore more established teaching and learning methods that are commonly used in experiential learning, such as service learning. As the literature indicates, service learning is a pedagogy that can enhance students’ awareness of a specific topic ( Ribeiro et al., 2023; Álvarez-Vanegas, Ramani, and Volante, 2024; Aramburuzabala, 2025). In the context of SARD, students can gain an understanding of sustainability by exploring the community through specific projects, which expose them to real-life phenomena related to sustainability within the Southeast Asian context.

Besides determining the pedagogical strategy, it is also expected that instructors will ensure, during the course of the study abroad program, that students are culturally aware of the customs relevant in the host country. Expectedly, it is also the instructors’ responsibility to ensure that they provide a safe place for students to express their concerns to counter mental health-related concerns of the students ( Campbell et al., 2022; Duffy, 2023; Li et al., 2025; Narayan et al., 2023; Vidourek et al., 2014; Zhang et al., 2024). As the literature suggests, one way instructors can address this concern is through the conduct of pre-departure orientations, which provide students with an insight into the cultural customs of the host country, as well as an introduction to the academic subjects that will be focused on at the host university. Therefore, whether the two institutions agree on a traditional teaching method for the students or are open to more alternative and collaborative learning methods, the students can be fully prepared for the different circumstances they may encounter.

It is also vital for instructors to consider re-entry orientations and assignments before departing for the student’s country of origin. As past studies show, there are multiple benefits in introducing these systems before returning, which, among many, include the countering of students struggling to integrate back into their societies ( Below et al., 2021; C. J. L. Campbell et al., 2015; Forbes-Mewett, 2011; Paras et al., 2019; Parmentier & Moore, 2016). Therefore, having a re-entry orientation allows students to voice their concerns, and instructors have the platform to reassure them. In addition, in relation to academic assessments, instructors can adopt different forms of assignments that cater to the unique dynamics of the study abroad program ( Gough, Janega, and Abu Dalo, 2018; Below, Nydegger, and Parmentier, 2021).

### 4.3. Establishing actively learning students

Courses on SARD can be challenging for IR students to grasp. The expectation of mastering a vast number of empirical cases related to the Southeast Asian region can be challenging for students if presented in a stagnant and less engaging manner. Therefore, the teaching and learning of an SARD course must ensure that it does so in a way that actively involves the students to help ignite their critical thinking and problem-solving skills, as expected in IR programs globally ( Arnold, 2015; Frueh et al., 2021; Ormes-Ganarin, 2014; She, 2021). One way to achieve this aim is by introducing experiential learning, which enables the establishment of an active learning atmosphere as students become the central actors in their own learning. As shown in the sub-sections previously, experiential learning through service-learning, for example, gives many of the learning responsibilities to the students.

To successfully adopt this experiential learning model in short-term study abroad programs, both instructors and participating students must assume several responsibilities to engage in this active learning approach fully. First relates to the costs associated with attending the program. As shown in the previous subsection, the length of programs varies significantly, and as recommended, it should not exceed a time length that would place an unnecessary financial burden on those participating in the program. Consequently, the first thing students may need to consider is fundraising projects. Whether conducted personally or collectively, fundraising programs help build a student’s creativity and oral communication skills ( Horta, Meoli, and Vismara, 2022; Madeo, 2022) as they step out of their comfort zones by engaging with the public.

Nevertheless, the idea of fundraising can take creative forms, such as engaging with different stakeholders. As the study abroad program fulfills the learning outcomes of the SARD course, students could consider engaging with stakeholders of interest in the Southeast Asian region, which may have connections to sustainability issues. If, for example, the experiential learning model selected takes the form of service-learning through a specific project on waste management in Southeast Asia, students may seek to engage with government stakeholders or non-governmental organizations to explore the forms of assistance those entities can provide to the participating students. Suppose a program involves a curriculum that builds upon a local or regional government’s sustainability program. In that case, students may be able to actively engage with local government stakeholders and explore whether the programs would receive financial support.

Besides the issue of costs, students’ responsibilities also relate to the cultures and academic subjects taught during the experiential learning program. In terms of culture, instructors have the responsibility to introduce the differences in cultural customs that are adopted in host countries. Nevertheless, students will need to undertake independent learning to delve deeper into the cultures, considering that, in the case of service-learning, for example, the students’ responsibilities would include communicating directly with society ( Brower, 2011; Anderson, Dore-Welch, and Johnson, 2019; Antal, 2024). Furthermore, in the case of the academic substantives, the pre-departure orientation provided by the instructors would only be able to touch the surface of the overall substantives and learning outcomes of the experiential learning. For example, suppose the study abroad program consists of a traditional teaching program facilitated by the host university. In that case, students will need to be prepared by actively asking questions or engaging in the facilitated discussions. For this, students need to undertake research before departing for the host university and prepare themselves for the program.

Considering the cultural and academic substance that students need to prepare themselves for, the students’ participation during the actual study abroad program would be filled with preparation. Therefore, whatever form experiential learning takes during the learning process, students are able to anticipate differences in cultures and actively engage with topics introduced during the learning process, due to the independent research and learning they undertook prior to their departure. As argued in the past literature on student-centered learning, the active participation of students in the learning process enables them to develop several lifelong skills and helps them cope with the uncertainties of the future ( Bhardwaj et al., 2025; Goodwin, 2024; Tang, 2023). As the SARD course is part of IR programs in many Asian universities, experiential learning methods also help establish IR graduates with the expected skills, such as critical thinking, teamwork, and effective communication.

## 5. Conclusion

Experiential learning through study abroad programs has the potential to achieve the learning outcomes of SARD courses in IR programs more engagingly and uniquely for students. As Below, Nydegger, and Parmentier mentioned in 2021, “A love of travel and cultural learning, together with a love of teaching, can provide a sound basis for developing study abroad programs with innovative ways to address IR curriculum and teaching” ( Below, Nydegger and Parmentier, 2021, p. 88). Such a prospect is indeed necessary in a study that engages with the topic of sustainability, which primarily requires an understanding of complex, interrelated topics related to emerging issues. However, as part of a broader IR program, the sustainability topics offered in SARD courses, mainly in Asian higher education institutions, often fall under the one-way teaching method that is commonly employed in many IR courses.

This study identifies the issue of teaching and learning sustainability topics within SARD courses as its main problem. The teaching of sustainability presents distinct challenges, as students need to witness firsthand the challenges that undermine sustainability practices across the region. However, with the existing teaching and learning systems, classroom teachings are unable to offer direct experiences of the world on sustainability issues due to their focus on empirical and theoretical explanations. Therefore, drawing from relevant literature on experiential learning and faculty-led short-term study abroad programs, this study bridges the idea of experiential learning, as proposed by Below, Nydegger, and Parmentier, and its potential to be adopted in the teaching and learning process of SARD courses. As argued by scholars in the past, experiential learning provides students with a unique learning environment, allowing them to observe firsthand the sustainability issues faced by communities and discuss potential solutions with their peers.

By adopting the mechanisms of experiential learning, faculty-led study abroad programs provide a feasible learning platform for students, considering several elements that the literature argues are important. Regarding the program’s outline, this study proposes several ideas for intertwining sustainability and Southeast Asia as the focus of the study abroad program. Despite sustainability being a minor topic within an SARD course, the program offers the option to select several emerging sustainability challenges in the Southeast Asian region, which helps ignite students’ curiosity and understanding of the region. These include issues such as haze pollution, IUUF, and climate change, which are unique to the Southeast Asian context. As the literature also shows, it is essential to incorporate topics related to cultural awareness within the substantive content, considering that the study abroad program will take place in a country with which students may not be familiar.

After selecting an appropriate theme for the study abroad program, the discussion then delves into the instructor’s responsibilities. Still related to the program outline, instructors are mandated to determine the timing of the program, which includes how long it will last and when the experiential learning program will be offered to the students. Related to this is the program’s destination, where instructors should be able to liaise with partnering universities and correspond with them regarding the proposal for a short-term study abroad program. After resolving the issues of time and destination, instructors should also consider the technical responsibilities of conducting this type of program, which typically require them to determine accommodation and transportation arrangements (although these technical tasks can be delegated through a third-party service). Lastly, instructors also have the responsibility of determining the best pedagogical strategy to be employed during the course of the program, as well as conducting pre- and re-entry orientations to prepare students well before their departure.

Third, this study zooms in on the students themselves. As participants in an experiential learning program, students also have certain expectations. At the first level, this includes cost-related responsibilities, in which students are expected to undertake fundraising projects and submit proposals to help finance their travels. Interestingly, as experiential learning can take the forms of service-learning, problem-based learning, and project-based learning, students would benefit from the independent learning that they will need to undertake before departing for the host country, as they delve deeper into the divergent cultural customs adopted in foreign countries, as well as research topics relevant to sustainability in the Southeast Asian context. The students’ active participation throughout the experiential learning course allows them to practice skills expected of IR graduates, including teamwork, critical thinking, and communication skills.

## Ethics statement

Ethics statement is not needed for this study.

## Data Availability

Data sharing is not applicable to this research as no data were generated or analysed.
