# Faculty-Led Study Abroad Programs and Learning Sustainability: A Southeast Asian Context

**DOI:** 10.12688/f1000research.178945.2

**Published:** 2026-06-05

**Authors:** Bama Andika Putra

**Affiliations:** 1 University of Bristol School of Sociology Politics and International Studies, Bristol, England, UK; 2 Universitas Hasanuddin Fakultas Ilmu Sosial dan Ilmu Politik, Makassar, South Sulawesi, Indonesia

**Keywords:** Study Abroad, Sustainability, Southeast Asia, International Relations, Pedagogy, Experiential Learning

## Abstract

As a widely offered course in International Relations (IR) programs, the teaching and learning of Southeast Asian Regional Dynamics (SARD) struggles to adopt a pedagogical strategy that counters stagnancy and passive learning. The problem is exacerbated when SARD courses discuss topics of sustainability in Southeast Asia, which obligates instructors to introduce complex empirical cases that may be difficult for students to grasp. As a means to ignite the students’ interests and allow a platform in which students can see directly the issues that defy sustainable development in the region, this study bridges the conception of experiential learning in SARD courses based on Below, Nydegger, and Parmentier’s study on short-term faculty-led study abroad programs, and looks at the prospects of its implementation into SARD courses. To make this argument, this conceptual narrative review explores the following ideas: The intertwining of sustainability and Southeast Asian contexts by exploring academic topics such as haze pollution, climate change, and illegal fishing within the context of Southeast Asia, as well as culturally-sensitive topics; acknowledgement of the instructors’ responsibilities within the context of program planning, technical arrangements, and the conduct of program orientations; and establishing active learning students through real-life learning mechanisms that elevate the students’ independent learning, research skills, communication skills, and critical thinking.

## 1. Introduction

Within the context of IR teaching and learning programs of higher education institutions, sustainability-related topics have always been an integral part of the curricula. In some universities, sustainability is offered as a single elective course; in others, it is part of a broader IR topic. For example, in a course on regional dynamics of IR, a course on SARD may include a topic on how sustainability has been achieved through regional collaborative efforts within the Association of Southeast Asian Nations (ASEAN), as well as the tensions that have arisen among the states in that region.

Nevertheless, the idea of sustainability differs from other analytical and conceptual frameworks within the study of IR. The United Nations Educational, Scientific, and Cultural Organization (UNESCO) announced the framework of Education for Sustainable Development as a global development agenda of ingraining sustainable development into the curricula of higher education institutions ( Glavič, 2020; Mochizuki & Vickers, 2024; Oe et al., 2022; UNESCO, 2024; Velempini & Maruatona, 2025). Consequently, UNESCO expects students to be able to develop the relevant knowledge and skills in relation to sustainable development, considering the high probability of the globe’s sustainability measures being in the students’ hands in a couple of years ( Elias & Bangkok, 2006; Glavič, 2020; Kohl et al., 2022). Therefore, in higher education institutions globally, there is growing concern about how best to teach sustainability to enhance students’ behaviors and skills in alignment with sustainable development.

However, in the case of IR teaching and learning, an issue arises immediately. IR classrooms, including those that cover sustainability topics, tend to focus on theoretical, conceptual, and empirical issues through traditional teaching methods in classrooms ( Bernstein, 2012; Lüdert, 2016; Campillo, Hernandez, and Cantano, 2021; Putra, 2025). While not denying the benefits of traditional teaching methods, the case of learning sustainability presents distinct challenges. Students need to see directly firsthand the issues that defy sustainable development to truly develop the behaviors and knowledge on the topic. As argued by many scholars in the past, this has been the reason why many universities globally adopt service-learning as a form of experiential learning, where there is a combination of both in-classroom teaching and community service ( Álvarez-Vanegas et al., 2024; Aramburuzabala, 2025; Aramburuzabala & Cerrillo, 2023; Ribeiro et al., 2023). Unfortunately, such a theme remains one that is unexplored within IR academia, considering that the focus on IR pedagogy as of late has been confined to student-centered learning approaches such as simulations, debates, discussions, and posters ( Duchin & Sherwood, 1990; Newmann & Twigg, 2000; Sasley, 2010; Sousa & Clark, 2019; Taylor, 2013; Wheeler, 2006).

The case is also evident in the teaching and learning processes of SARD courses. As a widely taught course in many Southeast Asian universities, SARD also struggles to adopt alternative pedagogies that replace the stagnant, passive learning that tends to prevail in classrooms. Therefore, although students gain greater knowledge of sustainability concerns in the Southeast Asian region by the end of the course, the course does not offer direct experience with global sustainability issues, as its focus tends to be on empirical and theoretical explanations. This is a concern, given the trend of higher education institutions undertaking systematic changes to orient research and education practices to sustainability ( Chou & Vun, 2025; Mensah, 2019; Wals, 2014). As a study in 2021 argued, “[…] universities as priority organisations and agents of change within the sphere of their social commitment” ( Prieto-Jiménez et al., 2021, p. 1).

Drawing on relevant literature on experiential learning and faculty-led study abroad programs, this conceptual narrative review argues that short-term study abroad programs are relevant as a means to supplement what is taught in SARD classes. Considering the complex nature of the topic, which comprises dynamic tensions among Southeast Asian states due to haze pollution, the difficulty of balancing economic performances and environmental protection, as well as challenges of climate change, this study perceives that students could benefit from a short-term abroad program that helps them work on a specific case related to sustainability. As argued by many scholars in the past, experiential learning provides a unique learning atmosphere for students as they delve into the center of the community to observe by themselves, dynamics related to the subjects being taught in classrooms ( Below et al., 2021; Blewitt et al., 2021; Kolb, 1984; Pipitone, 2018; Strange & Gibson, 2017). As a means to bridge the conception of experiential learning in the SARD courses, this study is informed by Amy Below, Amanda Nydegger, and Mary Jane Parmentier’s ‘Experiential Learning through Faculty-led Study Abroad Programs’ and looks to implement the program conceptions of the study into SARD courses, by reviewing how experiential learning can respond to the unique challenges encountered in the teaching of SARD and sustainability.

Experiential learning in IR is not new. However, it is less common within the specific context of SARD. In the past, scholars have argued that service learning offered as an experiential learning experience has been able to ignite the interests of students towards sustainable development in alignment with UNESCO’s Education for Sustainable Development ( Elias & Bangkok, 2006; Mochizuki & Vickers, 2024; Oe et al., 2022; UNESCO, 2024). This study aims to build on that by arguing how SARD courses can benefit from a short-term abroad program for those selecting the course, as a means to broaden the students’ horizon and look at the issues related to the nexus between sustainability and the Southeast Asian region beyond the lens of issues taking place within their country.

## 2. Learning sustainability: A literature review

Learning sustainability is not a simple task for the typical student. As Kleespies and Dierkes found in their 2022 study, social, economic, and environmental factors are interconnected in terms of sustainability ( Kleespies & Dierkes, 2022), leading to a multitude of issues affecting various actors. Therefore, in assessing alternative pedagogies in SARD courses, this study identifies the following discourses as relevant. They include a discourse on the barriers to learning about sustainability, the divergent methods adopted by instructors in the past, and the unique benefits of using service-learning in the context of understanding sustainable development. Taken together, the argument presented in this section is that existing scholarship has not demonstrated the applicability of experiential learning methods in region-specific cases, such as the SARD course, and that there is a need to adopt experiential methods to enhance students’ experiences.

The first discourse relevant to this study examines the numerous barriers to learning sustainability. Within this discourse, scholars acknowledge that sustainability is a complex topic, which leads to different variables affecting the success of the transfer of knowledge intended by instructors ( Grund et al., 2023; Parry & Metzger, 2023; Silva-Jean & Kneippb, 2024; Stam et al., 2023). As Stam, Ewjik, and Chan found in 2023, there is a complex relationship in the topic of ‘sustainability transitions,’ and a review is needed to assess whether the learning processes have led to more sustainable behaviors among students ( Stam, van Ewijk & Chan, 2023, p. 1). In a more general context, Perry and Metzger argued that the barriers to sustainable learning comprise multiple variables, such as “[…] disciplinary silos, […] high stakes assessments, and inadequate professional learning opportunities” ( Parry & Metzger, 2023, p. 1). The study is unique in that it centers on instructors’ concerns about their lack of preparation and competencies in relation to the teaching and learning processes of sustainable development.

Nevertheless, there is also considerable work examining other variables such as emotions, social learning, agency, and curriculum as the main barriers to sustainability learning ( Araneo, 2024; Grund et al., 2023; Koskela & Paloniemi, 2023; Silva-Jean & Kneippb, 2024). On the variable of emotions, for example, a 2023 study called for an “[…] educational practice where emotions can be experienced, expressed, and understood in a safe atmosphere” ( Grund, Singer-Brodowski and Büssing, 2023, p. 307), after arguing that emotions affect transformative learning processes. Looking at the sustainability issues students found interesting, Araneo’s study found that sustainability curricula can be tweaked to align with students’ interests across the general categories of sustainability-based sciences, relevant contexts, and hope for the future ( Araneo, 2024). Meanwhile, looking at the issue of sustainability learning from the lens of human agency theory, a study in 2023 argued the importance of reflecting on the concept of agency, and how such reflections can lead to “[…] countless actions in individual lives, communities, and local, national and global scenes can contribute to sustainability” ( Koskela & Paloniemi, 2023, p. 164).

Despite the rich theoretical and academic discussions on the barriers of sustainability learning, there have also been considerable studies made exploring divergent methods. Scholars have explored different approaches in teaching and learning sustainability, including community-based programs, problem-based learning, collaborative mechanisms, innovative pedagogies, as well as virtual experiences ( Bremner & Steed, 2025; Carrió Llach & Llerena Bastida, 2023; Gesthuizen et al., 2025; Nguyen et al., 2024; Shah et al., 2024). For example, studies on problem-based learning show that students can express more of their emotions due to the connection between the problem and real-life events ( Carrió Llach & Llerena Bastida, 2023; Nguyen et al., 2024).

In other cases, academics have explored various methods to establish practical approaches in teaching and learning sustainability. An example, through collaborative mechanisms with relevant institutions, a 2025 study concluded that knowledge on Sustainable Development Goals (SDGs) can be retrieved by bringing “[ …] students from other cultures and slightly different disciplines, with one being management and the other being practice-based, together to successfully demonstrate the use of an innovative method” ( Bremner & Steed, 2025, p. 171). Meanwhile, other studies, such as Howell, introduced the use of the ‘flipped classroom’ as a means to establish “[…] reflective and active learning” ( Howell, 2021, p. 1). In a more contemporary setting that incorporates modern technology, Gesthuizen, Tan, and Kidman found that virtual platforms are effective for designing sustainable virtual buildings and landscapes, as a means to establish “[…] future-thinking mindsets” ( Gesthuizen, Tan, and Kidman, 2025, p. 25).

Nevertheless, the majority of studies have focused on the more traditional methods of teaching and learning processes that help establish emotional connections with students’ surroundings. As noted in multiple studies on community service programs implemented in higher education institutions, these studies are united by the argument that these methods allow students to actively engage with their local communities and help “[…] fostering civic responsibility” ( Shah et al., 2024, p. 1).

The last discourse on service-learning encompasses most studies on sustainability learning methods. Through a service-learning pedagogical approach, students can enhance their knowledge of the SDGs and of practices that promote sustainable community development ( Brower, 2011; Ribeiro et al., 2023). Indeed, numerous studies in the past have established strong connections between service-learning and the eventual development of students’ awareness regarding the elements and dynamics of sustainability ( Pearce and Manion, 2016; Aramburuzabala and Cerrillo, 2023; Álvarez-Vanegas, Ramani, and Volante, 2024; Antal, 2024; Rodríguez-Zurita et al., 2025).

Specific studies would differ only in the points investigated in relation to service-learning. In a 2024 study, academics traced the processes leading to the substantial shift in the curriculum in sustainable development studies, which has now introduced more experiential learning methods ( Rodríguez-Zurita et al., 2025, p. 158). Meanwhile, a 2016 study highlighted the benefits of service-learning, using the example of community-based service-learning projects conducted at Virginia Tech ( Pearce & Manion, 2016). Service-learning, therefore, has established itself as one of the signature pedagogies for disseminating knowledge in sustainability studies, which many scholars have relied on in the past to raise students’ awareness ( Álvarez-Vanegas, Ramani, and Volante, 2024; Aramburuzabala, 2025; Narong, 2025).

But what about teaching and learning sustainability within a different curriculum, specifically SARD courses in IR? Unfortunately, the processes are not straightforward, as there has been no academic inquiry that specifically addresses the various contexts and challenges associated with raising awareness of sustainability topics and concerns among IR students in Southeast Asia. In a study made in 2024, scholars acknowledged the importance of educators as the leading actor that helps to determine a successful learning system for students, stating, “Educators play a pivotal role in implementation, and unless they are trained and incentivized and this is systemized, not only Service-Learning but also ESD may fail to transform learning environments” ( Álvarez-Vanegas, Ramani & Volante, 2024, p. 1). Similarly, Perry and Metzger argued, “[…] research shows that many practicing educators feel unprepared to help learners develop the competencies needed to forge more sustainable paths forward” ( Parry & Metzger, 2023, p. 1). Consequently, although this study recognizes the positive impact of service-learning in the context of learning sustainability, implementing it in SARD courses under IR programs is not straightforward. Educators should therefore be provided with guidelines for adopting such methods in specific contexts.

## 3. Enhancing Southeast Asian regional dynamics and sustainable development learning: Adopting experiential learning

Recognizing the lack of prior studies that address the ‘how’ of learning sustainability within the context of SARD courses under IR programs, this study advances the conception of experiential learning through short-term abroad programs, as proposed by Below, Nydegger, and Parmentier. Within the discourse of experiential learning, numerous studies in the past have argued how study abroad programs allows for greater achievement of a subject’s learning outcomes and advance students’ cross-cultural awareness ( Anderson et al., 2019; Bai et al., 2016; Edmunds & Shore, 2020; Giedt et al., 2015; Mule et al., 2018; Strange & Gibson, 2017; M. Tarrant & Lyons, 2012). As some scholars have noted, however, these faculty-led programs must be carefully formulated by instructors to achieve the expected outcomes, as positive outcomes are not automatic ( Parmentier and Moore, 2016; Mule, Audley, and Aloisio, 2018; Below, Nydegger, and Parmentier, 2021). Therefore, as this section aims to provide such a guideline, the ideas are inspired by the experiences of Below, Nydegger, and Parmentier in running short-term study abroad programs in Morocco and the Dominican Republic.

The first aims to outline a program, comprising the program design and curriculum. Perhaps the most significant task is to determine the destination and duration of a short-term abroad program as an experiential learning opportunity for students ( Below et al., 2021). Some questions to consider before announcing the program include whether students have access to scholarships from within or outside the university, and which term the program would be best offered. Regarding curriculum, the substantive focus would vary significantly between experiential learning programs. This should be based on the learning outcomes of a given course, to ensure alignment between what is being taught in the classroom and the actual program hosted abroad by the university. Nevertheless, as scholars have pointed out, there should be a balanced blend between subject-focused discussions and cross-cultural and interdisciplinary approaches in the program ( Tarrant, 2010; Tarrant and Lyons, 2012; Bell et al., 2016; Anderson, Dore-Welch, and Johnson, 2019; Below, Nydegger, and Parmentier, 2021). Other considerations should include the coverage of the discussions and background of the students ( Below et al., 2021; Mitchell & Maloff, 2016; Malewski & Phillion, 2009; Pipitone, 2018).

In addition to determining the substantive coverage of experiential learning, instructors should be aware of other responsibilities. From the onset, instructors should be able to answer several technical questions before initiating the program. In relation to the institution, it is essential to determine whether there are significant barriers and challenges within the university or department ( Howard and Keller, 2009; Below, Nydegger, and Parmentier, 2021). Once a program has taken shape, it is also essential for the instructors to determine whether there are potential partnering institutions abroad that could cooperate in the conduct of this short-term study abroad program ( Below, Nydegger, and Parmentier, 2021). One solution could be to ask the university’s partnerships department to compile a list of universities interested in cooperation, and then shortlist potential destinations after liaising with those institutions to assess their suitability for hosting students.

Another responsibility for the instructors concerns to the technical aspects of travel. Regarding accommodation, in-city transportation, and airport pickup, these technical aspects may be overwhelming for the instructors. Therefore, as suggested in several past studies, instructors could benefit from using a third-party service or a ‘provider’ that can help arrange the technical elements in a host country where an instructor may not be very familiar ( Eckert et al., 2016; Below, Nydegger, and Parmentier, 2021). A distinct benefit of using providers is that they can provide emergency support for the delegation and help the instructors by being the actors that “[…] knows the language, understands the legal systems, and can garner additional support and resources in the case of an emergency” ( Below, Nydegger, and Parmentier, 2021, p. 82).

Instructors are also burdened with multiple roles in the program. Not only are instructors expected to lead the effort in determining the program outline, but they are also expected to act as health professionals for students in times of emergency ( Below, Nydegger, and Parmentier, 2021). Instructors should therefore prepare themselves to take on roles to respond to mental health issues, considering how students may be exposed to new environments and may easily encounter cultural shocks ( Alkubaidi & Alzhrani, 2020; Demes & Geeraert, 2015; EL-ASRI et al., 2024; Tekel et al., 2025). One way to mitigate this, as recommended by Below, Nydegger, and Parmentier, is to conduct pre- and re-entry orientations. In both orientations, instructors have the opportunity to provide basic knowledge on what to expect throughout the program (subject and culture-related), as well as prepare the students to return to their everyday lives after the completion of the program ( Bai et al., 2016; Below et al., 2021; C. J. L. Campbell et al., 2015; Giedt et al., 2015; Paras et al., 2019).

The pedagogical strategy used throughout the short-term program would also need to be carefully determined by the instructors. As argued in the literature review section, multiple studies have supported the positive impact of service-learning on students’ learning experiences ( Aramburuzabala and Cerrillo, 2023; Álvarez-Vanegas, Ramani, and Volante, 2024; Aramburuzabala, 2025). Would there be a traditional in-class teaching experience offered in the program? Would the assignments be individual or group-based? What assignment activities would be given to the students? ( Below et al., 2021; Gough et al., 2018). These are all technical elements of the short-term abroad program that instructors need to be aware of.

Finally, several key points regarding students’ responsibilities need to be highlighted. Before their departure, one significant barrier that students will need to overcome is the cost of the program. As a means to enhance real-life skills ( Granato et al., 2024; Lien, 2007; Lien & Liu, 2010; Oduwaye et al., 2023), students would need to prepare their own funding to attend the program if a scholarship is not available ( Charlotte, 2019). Consequently, they will need to enhance their creative thinking by spreading out proposals or making joint fundraising programs to ensure that the cost barrier can be overcome.

Another issue students need to be aware of is preparing with the relevant materials for the program. In the pre-departure program, students must engage with the subject matter and culturally relevant topics covered to be prepared to participate in learning activities abroad. Meanwhile, throughout the program’s conduct, students are expected to be at the center of the learning process and to display active participation when hosted by another university ( Below, Nydegger, and Parmentier, 2021). Consequently, it is expected that students will remain active throughout the study abroad program and show respect for the unique cultural customs of the host country.

## 4. Faculty-led study abroad programs: Alternative methods in learning sustainability in the context of Southeast Asian regional dynamics

The ideas introduced by Below, Nydegger, and Parmentier will be integrated into the teaching and learning process of SARD courses within IR programs. In doing so, the following will be structured as follows. First, it provides a recommendation for a program’s outline, aiming to intertwine the fundamentals of sustainability with Southeast Asian contexts for an abroad program. Following this is an elaboration of the instructors’ responsibilities and what is expected from a participating student. By providing such guidelines, this study benefits instructors who seek to adopt experiential learning and integrate creative pedagogies with service-learning in their teaching and learning practices. In each subsection, the section explores relevant literature that helps build the case for the potential success of adopting the pedagogical proposals related to sustainability learning.

### 4.1 Outline of a Program: Ideas for intertwining sustainability and Southeast Asian contexts

The substantive coverage of an SARD course encompasses a wide range of empirical issues across Southeast Asia. A typical SARD course would begin by covering the history of the region, the complex political dynamics of the member states, efforts toward regional integration, and the contemporary challenges faced by Southeast Asian states. In relation to sustainability, therefore, SARD courses would only cover the topic as a minor class session or across several classes as an example of cooperation or as an illustration of barriers to regional engagement.

Consequently, it is essential to identify several potential sustainability-related topics in the Southeast Asian context that will serve as the basis for teaching and learning in the subjects offered in the short-term study abroad program. One of the first issues that instructors and students can explore is haze pollution in Southeast Asia. The haze problem is unique in the region, as it is a complex issue involving the roles of local, state, and regional actors ( Gan et al., 2021; Wang et al., 2023; Wangwongwatana, 2023; Y. Zhao et al., 2023). Indonesia, being one of the primary sources of haze in the region, has maintained the position that haze pollution also originates from other Southeast Asian states, which makes an Indonesia-specific solution unfair ( ASEAN, 2024a; Putra, 2024b; Varkkey, 2024). Regional organizations, such as ASEAN, have implemented numerous policies in the past to foster cooperation among Southeast Asian states, given the significant environmental and health impacts of haze pollution ( ASEAN, 2024a, 2024b, 2024c; Varkkey, 2024). Students could explore potential solutions to haze pollution, taking into account the complexities of negotiations over the past two decades.

Another topic that students can explore on the teaching and learning platforms of a study abroad program related to SARD is illegal, unreported, and unregulated fishing (IUUF). The Southeast Asian region is rich in sea-based resources, which the states in the region continue to exploit ( Fleck, 2024; Parameswaran, 2017; Resosudarmo & Kosadi, 2018; Schlieman, 2023). However, in recent years, there has been a significant rise in IUU fishing in Southeast Asian waters, which severely undermines the Southeast Asian states’ fisheries resources ( Chapsos et al., 2019; InvestSEA, 2023; IOJI, 2022; Noviansyah, 2018; Schlieman, 2023; Xiang, 2018). Besides the difficulty of counter IUUF in Southeast Asia, there is also the issue of overlapping Exclusive Economic Zones among states in the region, which has led to multiple episodes of fishing boats infiltrating the seas of other states to conduct fisheries activities ( BBC, 2016; Bhwana, 2020; IOJI, 2022; Juwana, 2014; Vu, 2020). Therefore, this environmental issue has a complex political context that necessitates thorough exploration to fully understand its nature and potential solutions.

Another topic that intertwines sustainability with the Southeast Asian regional context is climate change. Climate change is a global issue, not confined to any particular region. However, the dynamics in Southeast Asia are unique. First, Southeast Asian states are currently at a phase where industrialization is a trend among the government stakeholders, as they undertake measures to enhance the state’s economy by establishing industrialized systems ( de Pleijt & Frankema, 2025; Huq & Ichihashi, 2025; Kikuchi, 2018; Rasiah & Yun, 2009; X. Zhao, 2025). Consequently, this has led to practices that can be considered non-environmentally friendly, as state and private actors aim to achieve a particular target with little regard for the environment ( Rasiah & Yun, 2009; Zafar et al., 2020; Zafarullah & Mehnaz, 2025).

Within the context of Southeast Asia’s concerns over climate change are also the vast impacts that the region encounters. Southeast Asia primarily comprises littoral states and has direct boundaries with international seas. Consequently, in recent years, there has been a steady rise in sea levels directly linked to the global temperature increase ( IPCC, 2014; Lindsey, 2021). Another issue has been the increasing frequency of unpredictable weather in Southeast Asia, which has severely disrupted economic activities across the region’s states ( ADB, 2015; Prakash, 2018; Putra, 2024a; Suwandaru et al., 2024). Unfortunately, for typical IR students, it is challenging to grasp the impacts of climate change through a brief lecture in class. Through experiential learning, instructors can focus on the implications of climate change, allowing students to experience the issue firsthand, whether through direct observation or by exploring society and discussing its effects on local communities.

As mentioned in multiple studies in the past ( Anderson et al., 2019; Below et al., 2021), there is also a need for the substances offered to be balanced with the topic of cultural awareness. Given that students in a short-term study abroad program will visit a foreign country, considerable time should be invested in developing a curriculum that reflects the diversity of the host country’s culture and prepares them for what they will encounter. This includes issues that may be culturally sensitive, as well as the use of specific terminologies that should be avoided ( Bobel, Al Hinai, and Roslani, 2022; Turkson-Ocran et al., 2022; Narayan et al., 2023). In the context of SARD and sustainability, similar concerns warrant consideration. Local communities may be sensitive to some of the problems discussed in the context of sustainability, such as climate change, IUUF, or haze pollution. Therefore, in addition to a country’s basic cultural customs, a program should also be aware of the discourses relevant to the case studies to be assessed throughout the experiential learning experience.

### 4.2 Instructors’ responsibilities

After reviewing the potential themes for the study abroad program, instructors would need to take on several responsibilities to ensure the program’s success. Before the students’ actual departure, instructors must first determine the timing of the program ( Madden, McMillan, and Madden, 2019; Below, Nydegger, and Parmentier, 2021). This includes the program’s duration and the month in which it will be offered to students. As mentioned by Charlotte West, given the bearing costs, a short period (one or two weeks) should be sufficient to achieve the learning outcomes ( Charlotte, 2019). Meanwhile, the time offered within the academic year should be at the end of the SARD course. This allows students to gain a basic understanding of regional dynamics and helps them analyze cases presented during experiential learning.

As the responsible individual for the program, the instructor would then need to identify potential partner universities to conduct the short-term study abroad program. Instructors should acknowledge that their universities have established partnerships with other higher education institutions in the past, with some of these partnerships remaining active. Therefore, instructors should be able to identify host university options, taking into account that a particular higher education institution may have expertise and specialize in one of the themes the instructors wish to emphasize in the study abroad program. Nevertheless, an instructor should be able to weigh various considerations when determining the host country and university. For example, in a SARD course, instructors should first consider Southeast Asian Universities as the primary option if it is technically possible to make that long journey. As SARD courses are mainly offered in Asian higher education institutions, the bearing costs are not expected to be excessive. Instructors should address this point before the idea is introduced, as it will determine the proposal's viability depending on the enabling conditions.

Meanwhile, during the study abroad program, instructors are also responsible for the program's technical arrangements and the pedagogical strategy employed. On the former, the primary concern should be how instructors would arrange issues such as accommodations and transportation throughout the program. If the burden of this is unbearable, perhaps due to the high number of program participants, instructors should definitely explore the option of using providers ( Eckert et al., 2016; Below, Nydegger, and Parmentier, 2021). Despite the additional costs that must be paid, at least instructors can partially release themselves from the burden of technically arranging the trip once the delegations have arrived in the host country.

In doing so, a stronger emphasis can be placed on determining the most effective pedagogical strategy for the program. As mentioned in past studies, Below, Nydegger, and Parmentier stated, “[…] it is important to ensure our curricula are purposefully and holistically designed in order to have the positive impact we desire” ( Below, Nydegger, and Parmentier, 2021, p. 83). Instructors should then place a stronger emphasis on determining how the learning outcomes would be achieved by the conduct of the short-term study abroad program. To achieve this, it is essential to strike a balance between cultural substantives and interdisciplinary approaches in order to attain the desired outcomes ( Tarrant and Lyons, 2012; Bell et al., 2016; Anderson, Dore-Welch, and Johnson, 2019; Below, Nydegger, and Parmentier, 2021).

If instructors are looking to adopt a more interdisciplinary approach to their studies, perhaps examining innovative learning systems could provide the solution they need. As Below’s study argued, “[…] interdisciplinary approaches can facilitate rich conversations and learning experiences and attract students” ( Below, Nydegger and Parmentier, 2021, p. 83). Therefore, it would be beneficial to refer to past studies on alternative methods in teaching and learning sustainability, such as through problem-based learning, community-based learning, or quizzes ( Below et al., 2021; Bremner & Steed, 2025; Carrió Llach & Llerena Bastida, 2023; Howell, 2021; Nguyen et al., 2024; Shah et al., 2024). Meanwhile, instructors could also explore more established teaching and learning methods that are commonly used in experiential learning, such as service learning. As the literature indicates, service learning is a pedagogy that can enhance students’ awareness of a specific topic ( Ribeiro et al., 2023; Álvarez-Vanegas, Ramani, and Volante, 2024; Aramburuzabala, 2025). In the context of SARD, students can gain an understanding of sustainability by exploring the community through specific projects that expose them to real-life sustainability phenomena in the Southeast Asian context. Instructors should also be aware of the potential need of ethical clearance from their host university, if it is needed in the process of researching or conducting projects on sensitive topics.

Besides determining the pedagogical strategy, it is also expected that instructors will ensure, during the course of the study abroad program, that students are culturally aware of the customs relevant in the host country. Expectedly, it is also the instructors’ responsibility to ensure that they provide a safe place for students to express their concerns to counter mental health-related concerns of the students ( Campbell et al., 2022; Duffy, 2023; Li et al., 2025; Narayan et al., 2023; Vidourek et al., 2014; Zhang et al., 2024). As the literature suggests, one way instructors can address this concern is by conducting pre-departure orientations, which provide students with insight into the host country's cultural customs and an introduction to the academic subjects that will be focused on at the host university. Therefore, whether the two institutions agree on a traditional teaching method for students or are open to alternative and collaborative learning methods, students can be fully prepared for the different circumstances they may encounter.

It is also vital for instructors to consider re-entry orientations and assignments before departing for the student’s country of origin. As past studies show, there are multiple benefits in introducing these systems before returning, which, among many, include the countering of students struggling to integrate back into their societies ( Below et al., 2021; C. J. L. Campbell et al., 2015; Forbes-Mewett, 2011; Paras et al., 2019; Parmentier & Moore, 2016). Therefore, having a re-entry orientation allows students to voice their concerns, and instructors have the platform to reassure them. In addition, regarding academic assessments, instructors can adopt various of assignment formats that cater to the unique dynamics of the study abroad program ( Gough, Janega, and Abu Dalo, 2018; Below, Nydegger, and Parmentier, 2021).

### 4.3 Establishing actively learning students

Courses on SARD can be challenging for IR students to grasp. The expectation of mastering a large number of empirical cases related to the Southeast Asian region can be challenging for students when presented in a static and less engaging manner. Therefore, the teaching and learning of an SARD course must ensure that it does so in a way that actively involves the students to help ignite their critical thinking and problem-solving skills, as expected in IR programs globally ( Arnold, 2015; Frueh et al., 2021; Ormes-Ganarin, 2014; She, 2021). One way to achieve this aim is to introduce experiential learning, which fosters an active learning environment as students become the central actors in their own learning. As shown in the previous subsections, experiential learning through service-learning, for example, assigns many of the learning responsibilities to students.

To successfully adopt this experiential learning model in short-term study abroad programs, both instructors and participating students must assume several responsibilities to fully engage in this active learning approach. First relates to the costs associated with attending the program. As shown in the previous subsection, program lengths vary significantly, and, as recommended, they should not exceed a duration that would impose an unnecessary financial burden on participants. Consequently, the first thing students may need to consider is fundraising projects. Whether conducted personally or collectively, fundraising programs help build a student’s creativity and oral communication skills ( Horta, Meoli, and Vismara, 2022; Madeo, 2022) as they step out of their comfort zones by engaging with the public.

Nevertheless, fundraising can take creative forms, such as engaging with diverse stakeholders. As the study abroad program fulfills the learning outcomes of the SARD course, students could consider engaging with stakeholders of interest in Southeast Asia who may be connected to sustainability issues. If, for example, the selected experiential learning model takes the form of service-learning through a specific project on waste management in Southeast Asia, students may seek to engage with government stakeholders or non-governmental organizations to explore the forms of assistance those entities can provide to participating students. Suppose a program involves a curriculum that builds upon a local or regional government’s sustainability program. In that case, students may be able to actively engage with local government stakeholders and explore whether the programs would receive financial support.

Beyond costs, students’ responsibilities also relate to the cultures and academic subjects taught in the experiential learning program. In terms of culture, instructors are responsible for introducing differences in cultural customs adopted in host countries. Nevertheless, students will need to undertake independent learning to delve deeper into the cultures, given that, in the case of service-learning, for example, students’ responsibilities would include communicating directly with society ( Brower, 2011; Anderson, Dore-Welch, and Johnson, 2019; Antal, 2024). Furthermore, in the case of the academic substantives, the pre-departure orientation provided by the instructors would only be able to touch the surface of the overall substantives and learning outcomes of the experiential learning. For example, suppose the study abroad program consists of a traditional teaching program facilitated by the host university. In that case, students will need to be prepared by actively asking questions or engaging in the facilitated discussions. For this, students need to undertake research before departing for the host university and prepare themselves for the program.

Considering the cultural and academic preparation students need, their participation during the actual study abroad program would be well-prepared. Therefore, whatever form experiential learning takes, students are able to anticipate cultural differences and actively engage with topics introduced during the learning process, due to the independent research and learning they undertook prior to their departure. As argued in prior literature on student-centered learning, students' active participation in the learning process enables them to develop several lifelong skills and helps them cope with future uncertainties ( Bhardwaj et al., 2025; Goodwin, 2024; Tang, 2023). As the SARD course is part of IR programs in many Asian universities, experiential learning methods also help establish IR graduates with the expected skills, such as critical thinking, teamwork, and effective communication.

## 5. Conclusion

Experiential learning through study abroad programs has the potential to achieve the learning outcomes of SARD courses in IR programs in a more engaging and distinctive way for students. As Below, Nydegger, and Parmentier mentioned in 2021, “A love of travel and cultural learning, together with a love of teaching, can provide a sound basis for developing study abroad programs with innovative ways to address IR curriculum and teaching” ( Below, Nydegger and Parmentier, 2021, p. 88). Such a prospect is indeed necessary in a study that engages with sustainability, which primarily requires an understanding of complex, interrelated issues. However, as part of a broader IR program, the sustainability topics offered in SARD courses, mainly at Asian higher education institutions, often follow the one-way teaching method commonly employed in many IR courses.

This conceptual narrative review identifies the issue of teaching and learning sustainability topics within SARD courses as its main problem. Teaching sustainability presents distinct challenges, as students need to witness firsthand the issues undermining sustainability practices across the region. However, within existing teaching and learning systems, classroom teaching cannot offer direct experiences of the world regarding sustainability issues because it focuses on empirical and theoretical explanations. Therefore, drawing on relevant literature on experiential learning and faculty-led short-term study abroad programs, this study bridges the concept of experiential learning, as proposed by Below, Nydegger, and Parmentier, and its potential for adoption in the teaching and learning process of SARD courses. As scholars have argued in the past, experiential learning provides students with a unique learning environment, allowing them to observe firsthand the sustainability issues faced by communities and discuss potential solutions with their peers.

Through the pedagogical proposal offered in this study, faculty-led study abroad programs, as mechanisms of experiential learning, potentially provide a feasible learning platform for students, considering several elements that the literature argues are important. Regarding the program’s outline, this study proposes several ideas for intertwining sustainability and Southeast Asia as the focus of the study abroad program. Although sustainability is a minor topic in an SARD course, the program offers the option to select several emerging sustainability challenges in Southeast Asia, which helps ignite students’ curiosity and understanding of the region. These include issues such as haze pollution, IUUF, and climate change, which are unique to the Southeast Asian context. As the literature also shows, it is essential to incorporate cultural awareness topics into the substantive content, given that the study abroad program will take place in a country with which students may not be familiar.

After selecting an appropriate theme for the study abroad program, the discussion then delves into the instructor’s responsibilities. Still related to the program outline, instructors are mandated to determine the timing of the program, including how long it will last and when it will be offered to students. Related to this is the program’s destination, where instructors should be able to liaise with partnering universities and correspond with them regarding the proposal for a short-term study abroad program. After resolving the issues of timing and destination, instructors should also consider the technical responsibilities of conducting this type of program, which typically require them to arrange accommodations and transportation (although these tasks can be delegated to a third-party service). Lastly, instructors are responsible for determining the best pedagogical strategy to be employed throughout the program, as well as for conducting pre- and re-entry orientations to prepare students well before their departure.

Third, this study zooms in on the students themselves. As participants in an experiential learning program, students also have certain expectations. At the first level, this includes cost-related responsibilities, in which students are expected to undertake fundraising projects and submit proposals to help finance their travels. Interestingly, as experiential learning can take the forms of service-learning, problem-based learning, and project-based learning, students would benefit from the independent learning that they will need to undertake before departing for the host country, as they delve deeper into the divergent cultural customs adopted in foreign countries, as well as research topics relevant to sustainability in the Southeast Asian context. The students’ active participation throughout the experiential learning course allows them to practice skills expected of IR graduates, including teamwork, critical thinking, and communication skills.

## Ethics statement

Ethics statement is not needed for this study.

## Data Availability

Data sharing is not applicable to this research as no data were generated or analysed.
