# Supplementary material for: Faculty-Led Study Abroad Programs and Learning Sustainability: A Southeast Asian Context
Source: F1000Res. 2026 Jun 5;15:499. Originally published 2026 Apr 10. [Version 2] doi: 10.12688/f1000research.178945.2 (PMC13554790; doi:10.12688/f1000research.178945.2)
Supplement: Peer review history

# Reviewer report: Reviewer response for version 2

**DOI:** 10.5256/f1000research.202356.r500904

**Published:** 2026-07-29

**Authors:** Rashed Ahmed

**Affiliations:** 1 Pharmaceutical technology, North South University, Dhaka, Dhaka Division, Bangladesh

I have carefully evaluated the revised manuscript entitled **"Faculty-Led Study Abroad Programs and Learning Sustainability: A Southeast Asian Context."** The manuscript addresses a timely and relevant topic by exploring how faculty-led study abroad programs can be integrated into International Relations (IR) curricula to enhance sustainability education within the context of Southeast Asian Regional Dynamics (SARD). The revised version demonstrates noticeable improvements over the previous submission, particularly by clearly positioning the manuscript as a conceptual narrative review, including methodological notes on literature selection, moderating some conclusions, and clarifying ethical considerations.

 The manuscript provides a broad overview of the literature on sustainability education, experiential learning, service learning, and faculty-led study abroad programs, and then proposes a conceptual framework for implementing these approaches within SARD courses. The discussion is generally well organized and supported by an extensive reference list, and the proposed framework offers practical value for educators interested in curriculum innovation. The topic is relevant, and the manuscript has the potential to contribute to the growing literature on Education for Sustainable Development (ESD) within higher education.

 However, despite these improvements, several issues still limit the manuscript in its current form.

 First, although methodological transparency has improved, the literature review methodology remains relatively limited. The manuscript identifies itself as a conceptual narrative review, but the procedures for identifying, selecting, and synthesizing the literature are only briefly described. A more detailed explanation of the literature search strategy, the databases consulted, the search terms, the publication time frame, the inclusion and exclusion criteria, and the rationale for selecting the cited studies would improve transparency, reproducibility, and readers' confidence in the comprehensiveness of the review.

 Second, the manuscript remains predominantly descriptive. While numerous studies are summarized, there is comparatively little critical evaluation of the existing evidence. The review would benefit from a more analytical synthesis that compares findings across studies, identifies conflicting evidence, discusses methodological strengths and weaknesses of previous research, evaluates the quality of available evidence, and highlights important knowledge gaps requiring future investigation.

 Third, although the review includes a substantial number of recent references, the integration of the literature could be improved. Several sections present sequential summaries of individual studies rather than synthesizing the evidence into broader conceptual insights. Organizing the discussion around major themes, areas of agreement, controversies, and unresolved issues would substantially strengthen the scholarly contribution of the review.

 Fourth, several recommendations presented throughout the manuscript appear stronger than the currently available evidence supports. The proposed pedagogical framework is logically developed, but many anticipated educational benefits are inferred from the broader experiential learning literature rather than being supported by direct empirical evidence from SARD-specific implementations. The conclusions should therefore more explicitly acknowledge the conceptual nature of the framework and emphasize the need for future empirical validation through pilot implementations and educational assessment.

 Fifth, the manuscript gives relatively little attention to the limitations of faculty-led study abroad programs themselves. Potential challenges, including financial accessibility, unequal participation opportunities, institutional resource limitations, visa restrictions, safety considerations, logistical complexity, and sustainability of repeated program implementation, deserve greater discussion to provide a more balanced perspective.

 Sixth, the discussion of assessment remains underdeveloped. While the manuscript proposes several educational activities, it provides limited consideration of how learning outcomes, sustainability competencies, intercultural development, or long-term educational impacts would be measured. Including recommendations for assessment tools, evaluation frameworks, or measurable learning outcomes would considerably strengthen the practical value of the proposed model.

 Seventh, the manuscript would benefit from a more thorough discussion of transferability and generalizability. The proposed framework is developed specifically for SARD courses, yet the manuscript does not sufficiently discuss how institutional diversity, national educational policies, cultural contexts, or resource availability across Southeast Asia may influence implementation.

 Eighth, although ethical considerations have been clarified compared with the previous version, the discussion remains relatively brief. More consideration should be given to ethical issues associated with international experiential learning, including community engagement, reciprocity with host institutions, responsible research practices, cultural sensitivity, participant welfare, and minimizing unintended impacts on local communities.

 Ninth, although the manuscript cites a wide range of studies, the review could better distinguish between evidence derived from empirical educational research and recommendations based primarily on conceptual reasoning. Doing so would help readers clearly understand which recommendations are evidence-based and which remain theoretical propositions.

 Tenth, the manuscript lacks a concise section outlining future research priorities. Identifying specific research directions, such as longitudinal evaluation of learning outcomes, comparative studies across universities, assessment of intercultural competencies, cost-effectiveness analyses, and implementation studies, would increase the review's usefulness for future researchers.

 Finally, although the overall organization has improved substantially, some sections remain repetitive, particularly where similar concepts regarding experiential learning, instructor responsibilities, student engagement, and service learning are revisited across multiple sections. Condensing repetitive discussions, improving transitions between sections, and streamlining lengthy paragraphs would improve readability and strengthen the overall flow of the manuscript.

 Overall, this revised manuscript addresses an important and emerging topic in sustainability education and international higher education pedagogy. The conceptual framework is interesting and has potential value for educators; however, additional revisions are still required to strengthen methodological transparency, improve critical synthesis of the literature, provide a more balanced discussion of implementation challenges and limitations, moderate conclusions where empirical evidence remains limited, enhance discussion of assessment and future research, and improve the overall coherence and scholarly rigor of the review before indexing.

**Recommendation: Major Revision.**

Is the review written in accessible language?

Partly

Are all factual statements correct and adequately supported by citations?

Partly

Are the conclusions drawn appropriate in the context of the current research literature?

Partly

Is the topic of the review discussed comprehensively in the context of the current literature?

Partly

Reviewer Expertise:

Pharmaceutical Sciences; Clinical Pharmacy; Pharmacology; Drug Discovery; Pharmaceutical Analysis; Drug Regulatory Affairs; Public Health; Artificial Intelligence in Healthcare; Sustainability; Environmental Health; Higher Education Research.

I confirm that I have read this submission and believe that I have an appropriate level of expertise to confirm that it is of an acceptable scientific standard, however I have significant reservations, as outlined above.

## References

[1] : Exploring ChatGPT Usage in Higher Education: Patterns, Perceptions, and Ethical Implications Among University Students. *JOURNAL OF DIGITAL LEARNING AND DISTANCE EDUCATION* .2024;3(6) : 10.56778/jdlde.v3i6.363 1122-1131 10.56778/jdlde.v3i6.363

[2] : Race, Power, Privilege, A Critical Analysis of Systematic Racism. *MANDALIKA : Journal of Social Science* .2026;4(1) : 10.56566/mandalika.v4i1.441 27-36 10.56566/mandalika.v4i1.441

[3] : Exploring Barriers to Technology Integration in Nursing Education: An Unveiling through a Qualitative Study.2025; 10.31235/osf.io/wemr3_v1 10.31235/osf.io/wemr3_v1

# Reviewer report: Reviewer response for version 2

**DOI:** 10.5256/f1000research.202356.r491180

**Published:** 2026-06-13

**Authors:** Rubén Baena-Navarro

**Affiliations:** 1 Universidad de Córdoba, Montería, Colombia

I thank the author for revising the manuscript and for providing detailed responses to the previous round of review. The revised version of “Faculty-Led Study Abroad Programs and Learning Sustainability: A Southeast Asian Context” represents a substantive improvement over the original submission. In particular, I appreciate that the article is now more clearly framed as a conceptual narrative review, that the conclusions have been partially moderated, that institutional enabling conditions are more explicitly acknowledged, and that an initial ethical consideration has been added regarding the possible need for ethical clearance when students conduct research or projects on sensitive topics.

 Overall, I consider that the manuscript has moved toward a clearer and more defensible contribution to International Relations pedagogy, particularly in relation to the teaching of sustainability in Southeast Asian Regional Dynamics courses. The manuscript remains relevant because it addresses a specific curricular problem: the difficulty of teaching complex sustainability issues—such as haze pollution, illegal, unreported and unregulated fishing, and climate change—through predominantly lecture-based approaches. The article also retains practical value by organizing the proposal around three main components: the selection of regionally relevant sustainability topics, instructors’ responsibilities, and students’ active participation.

 My remaining reservations are now more limited than in the first round. I do not think the manuscript needs to be transformed into an empirical study or a systematic review. However, several focused revisions would still make the conceptual contribution more transparent, traceable, and scientifically proportionate.

 First, the clarification of the manuscript as a “conceptual narrative review” addresses an important part of the previous concern, but the article would still benefit from a short methodological note explaining how the narrative review was constructed. The manuscript does not need a PRISMA protocol or a systematic-search design. However, it should briefly indicate which bodies of literature were integrated, what general criteria guided the selection of key sources, and how the author moved from those sources to the proposed pedagogical components. This would strengthen the auditability of the argument without changing the conceptual nature of the paper.

 Second, the author’s response to the concern about traceability is reasonable, but still not fully sufficient. Section 4 is now better organized into three subthemes, which improves readability. Nevertheless, the relationship between the literature reviewed and the specific component of the proposal remains partly implicit. I recommend adding a brief table or conceptual diagram linking each component of the proposal—topic selection, pre-departure orientation, instructor responsibilities, student responsibilities, pedagogical strategies, and re-entry orientation—to the strands of literature that support it and to the type of inference being made. This would not impose an excessive methodological burden, but it would allow readers to verify more clearly how the documentary review is translated into specific pedagogical recommendations for Southeast Asian Regional Dynamics courses.

 Third, the added reference to ethical clearance is a useful improvement, but the ethical framing could still be strengthened. The manuscript correctly indicates that the present conceptual review did not generate or analyze primary data. However, the future pedagogical implementation being proposed may involve engagement with communities, local actors, and culturally sensitive environments. I therefore suggest distinguishing explicitly between the absence of a need for ethical approval for the present article and the ethical conditions required for future implementation. These conditions should include cultural preparation, consent or contextual authorization when appropriate, coordination with host partners, prevention of extractive engagement, student safety protocols, and referral mechanisms for mental-health or culture-shock-related concerns.

 Fourth, the conclusions have been moderated compared with the previous version, but some formulations still risk suggesting a stronger level of effectiveness than the design can demonstrate. Since the article does not implement a program, compare cohorts, measure learning outcomes, or provide original empirical evidence, claims about the impact of faculty-led study abroad programs should remain conditional. I recommend replacing formulations that imply direct achievement of competences with expressions such as “may support,” “could contribute,” “may provide opportunities,” or “requires future empirical assessment.” This is a matter of claim calibration rather than a need for new data.

 Fifth, the scope of applicability should be made more explicit. The manuscript now recognizes that institutional partnerships and feasibility are relevant conditions, but it still appears somewhat optimistic regarding costs and students’ ability to finance participation through fundraising. The proposal would be stronger if it clarified that implementation depends on scholarships, subsidies, active partnerships with host universities, administrative support, insurance, safety protocols, instructor capacity, and equitable access criteria. This would prevent the proposal from being interpreted as universally applicable or as relying primarily on students’ financial initiative.

 Sixth, I recommend refining the description of the instructor’s role in relation to mental health and emergencies. It is appropriate for the manuscript to acknowledge that international programs may generate stress, culture shock, or support needs. However, instructors should not be framed as “health professionals” or as substitutes for qualified professionals. A more precise formulation would indicate that instructors should be familiar with emergency protocols, provide first-line support, coordinate with institutional services, and refer students to qualified professionals when necessary. This distinction would better protect both students and instructors.

 Finally, I do not identify concerns regarding equations, quantitative tables, analytical figures, or numerical results, because the manuscript does not include those components and its conceptual nature does not require them. Precisely for that reason, a conceptual traceability table would be sufficient to improve internal verifiability. I also recommend checking minor issues of terminological and editorial consistency, especially the use of IUU/IUUF, some cited author names—for example, Parry/Metzger and van Ewijk—and a few grammatical formulations that could be adjusted to improve academic precision.

 I consider that the author has responded reasonably to the main concerns raised in the first review round. The remaining issues are important but focused, and they can be addressed through clarification, moderation of language, targeted improvement of conceptual traceability, and a more precise delimitation of implementation conditions. My editorial recommendation is therefore approval with reservations, subject to minor revisions aimed at strengthening methodological transparency, ethical delimitation, proportionality of conclusions, and the applicability conditions of the proposal.

Is the review written in accessible language?

Yes

Are all factual statements correct and adequately supported by citations?

Partly

Are the conclusions drawn appropriate in the context of the current research literature?

Partly

Is the topic of the review discussed comprehensively in the context of the current literature?

Partly

Reviewer Expertise:

NA

I confirm that I have read this submission and believe that I have an appropriate level of expertise to confirm that it is of an acceptable scientific standard, however I have significant reservations, as outlined above.

# Reviewer report: Reviewer response for version 1

**DOI:** 10.5256/f1000research.197391.r475159

**Published:** 2026-04-29

**Authors:** Rany Sam

**Affiliations:** 1 National University of Battambang, Battambang, Cambodia

The article argues that traditional lecture-based teaching in Southeast Asian Regional Dynamics (SARD) courses fails to convey the complexity of sustainability and proposes short-term, faculty-led study abroad programs as an experiential learning approach to enhance student engagement, skills, and real-world understanding. It clearly identifies a pedagogical gap and offers a structured, practice-oriented framework grounded in relevant regional issues, including haze pollution, illegal fishing, and climate change. The manuscript is well-written and conceptually valuable, but it is only partially comprehensive and lacks full methodological robustness. Major issues that must be addressed for scientific soundness: (1) clearly define the article as a conceptual/narrative review rather than an empirical “study”; (2) add a transparent methodology explaining literature selection, scope, and synthesis; and (3) moderate claims of effectiveness, reframing conclusions as plausible proposals rather than demonstrated outcomes. Without these revisions, the evidentiary basis and validity of the conclusions remain insufficiently supported.

 Further improvements are needed to strengthen rigor and applicability. The literature review is relevant but insufficiently systematized; the authors should explicitly map how specific strands of literature inform each component of the proposed framework (e.g., via a table or diagram). Claims about feasibility and impact should be better supported or clearly qualified, particularly given the absence of empirical testing. Additionally, the manuscript should more explicitly define contextual limitations (e.g., institutional capacity, funding, partnerships) to avoid overgeneralization, and include a brief discussion of ethical considerations in community-based experiential learning (e.g., cultural sensitivity, non-extractive engagement). These latter points are important but secondary; the core mandatory revisions remain methodological transparency, conceptual clarity, and claim calibration. Addressing these would make the article a credible and useful contribution to IR pedagogy.

Is the review written in accessible language?

Yes

Are all factual statements correct and adequately supported by citations?

Yes

Are the conclusions drawn appropriate in the context of the current research literature?

Yes

Is the topic of the review discussed comprehensively in the context of the current literature?

Yes

Reviewer Expertise:

Higher education and social sciences

I confirm that I have read this submission and believe that I have an appropriate level of expertise to confirm that it is of an acceptable scientific standard.

# Reviewer report: Reviewer response for version 1

**DOI:** 10.5256/f1000research.197391.r475163

**Published:** 2026-04-24

**Authors:** Rubén Baena-Navarro

**Affiliations:** 1 Universidad de Córdoba, Montería, Colombia

Thank you for the opportunity to review this manuscript. The article addresses a relevant and well-defined pedagogical problem: how to strengthen the teaching of sustainability in *Southeast Asian Regional Dynamics* (SARD) courses within International Relations programs by moving beyond predominantly lecture-based and weakly experiential teaching models. The manuscript proposes adapting the *faculty-led study abroad* approach to the SARD context, with emphasis on three main components: the selection of sustainability topics relevant to Southeast Asia, the responsibilities of instructors, and the active role of students. Its main contribution is to offer a conceptual and practice-oriented proposal for thinking about more situated, active, and interdisciplinary ways of teaching sustainability in this curricular niche.

 Among its genuine strengths, the manuscript identifies a specific gap at the intersection of International Relations pedagogy, sustainability education, and Southeast Asian regional studies. It also goes beyond defending experiential learning in abstract terms by attempting to translate that approach into concrete curricular design orientations. In addition, the proposal is anchored in regionally recognizable issues such as transboundary haze, illegal, unreported and unregulated fishing, and climate change, which improves both disciplinary relevance and pedagogical usefulness. The article therefore has merit as a conceptual contribution with practical teaching implications.

 That said, I believe the manuscript still requires substantial revision in order to become fully scientifically sound. My first critical concern relates to the definition of the article type and to the traceability of its knowledge-construction strategy. The text presents itself as a “study,” but in practice it functions as a narrative review combined with a conceptual pedagogical design proposal. That is not a problem in itself; the problem is that this nature is not stated clearly enough. The manuscript does not report literature selection criteria, temporal scope, logic of source integration, or the procedure through which it moves from prior literature to recommendations for SARD. This limits the auditability of the paper and makes it difficult to assess precisely the evidentiary basis of its main contribution. This point should be addressed as an essential requirement through a short but explicit methodological clarification. The paper does not need to be turned into a systematic review, but it does need to provide the minimum transparency required for readers to understand how the proposal was built.

 My second critical concern involves the proportionality between evidence and conclusions. The manuscript plausibly argues that short-term study abroad programs could enrich sustainability learning in SARD courses. However, in the conclusion and in several parts of the discussion, the language at times approaches a claim of demonstrated feasibility or effectiveness. Since the article does not implement a program, does not report intervention outcomes, does not compare cohorts, and does not provide original empirical evidence on student learning, that degree of closure goes beyond what the design can actually support. I do not see this as a fatal flaw in the idea itself, but rather as a problem of claim calibration. To make the article scientifically stronger, the conclusions should be reformulated as a conceptually grounded and plausible proposal rather than as an already validated instructional platform.

 My third observation concerns the comprehensiveness of the review. The manuscript includes recent and relevant references and demonstrates familiarity with current debates on sustainability education, service-learning, and study abroad. However, the literature discussion is not fully exhaustive and is not analytically systematized to a sufficient degree. In particular, the transition from barriers to sustainability learning, to alternative pedagogies, to the adoption of the Below, Nydegger, and Parmentier framework depends more on a reasonable argumentative bridge than on an explicitly traceable synthesis. It would be very helpful to include a correspondence table or conceptual diagram showing which strands of the literature support each component of the proposal: topic selection, pre-departure orientation, instructor responsibilities, student responsibilities, and possible pedagogical strategies during the program. This would substantially strengthen the manuscript’s internal coherence.

 My fourth observation concerns the delimitation of the context of applicability. The article proposes this model as a useful alternative for SARD courses in Asian universities and even suggests that intra-regional mobility may reduce costs. However, the proposal relies on strong institutional assumptions: active international partnerships, instructor capacity for cross-border coordination, access to funding or realistic fundraising opportunities, and adequate logistical support. The text mentions some of these factors, but it does not clearly delimit that the viability of the proposal depends on such enabling conditions. In my view, this does not require redesigning the paper, but it does require making the implementation assumptions explicit so that readers do not interpret the proposal as universally applicable.

 My fifth important observation relates to the ethical and relational framing of the proposal. The manuscript states that no ethics statement is needed, which is understandable if the article is read strictly as a non-empirical piece without data collection. However, because the paper recommends forms of learning that may involve engagement with communities, local actors, and culturally sensitive environments, it would be valuable to distinguish between research ethics and ethics of pedagogical implementation. A brief expansion on this point would strengthen the manuscript, particularly regarding cultural preparation, relationships with host communities, and the prevention of extractive or instrumentalizing approaches to the local context.

 Regarding the specific form questions, I consider the topic to be discussed partly in the context of the current literature: the coverage is relevant, current, and sufficient to support an initial proposal, but it is not yet fully comprehensive or methodologically transparent. I also consider the factual statements to be partly correct and adequately supported by citations: overall, the manuscript cites extensively, but some broader claims about applicability, expected benefits, or implementation conditions rely more on reasonable inference than on support directly demonstrated for the SARD context. The review is written in accessible language: the text is clear, readable, and generally well organized. Finally, the conclusions are partly appropriate in relation to the current research literature: the overall argumentative direction is valid, but the final wording should be moderated so as not to imply empirical validation where the article provides conceptual grounding only.

 I believe the manuscript contains a valuable idea and a potentially useful contribution to International Relations pedagogy, but it still requires substantive revision before it can be considered fully scientifically sound. My editorial recommendation is major revisions. The priority actions that would most improve the paper are: (1) clearly define the manuscript as a conceptual or narrative-review-based contribution; (2) add a brief methodological note on literature selection and integration; (3) include a traceability mechanism linking literature to the proposed framework; (4) moderate the scope of the conclusions; and (5) specify more clearly the institutional and ethical conditions under which the proposal would be applicable.

Is the review written in accessible language?

Yes

Are all factual statements correct and adequately supported by citations?

Partly

Are the conclusions drawn appropriate in the context of the current research literature?

Partly

Is the topic of the review discussed comprehensively in the context of the current literature?

Partly

Reviewer Expertise:

NA

I confirm that I have read this submission and believe that I have an appropriate level of expertise to confirm that it is of an acceptable scientific standard, however I have significant reservations, as outlined above.

# Response

**Published:** 2026-05-23

**Authors:** bama andika putra

**Affiliations:** University of Bristol School of Sociology Politics and International Studies, Bristol, England, UK

Comment 1:

 My first critical concern relates to the definition of the article type and to the traceability of its knowledge-construction strategy. The text presents itself as a “study,” but in practice it functions as a narrative review combined with a conceptual pedagogical design proposal. That is not a problem in itself; the problem is that this nature is not stated clearly enough. The manuscript does not report literature selection criteria, temporal scope, logic of source integration, or the procedure through which it moves from prior literature to recommendations for SARD. This limits the auditability of the paper and makes it difficult to assess precisely the evidentiary basis of its main contribution. This point should be addressed as an essential requirement through a short but explicit methodological clarification. The paper does not need to be turned into a systematic review, but it does need to provide the minimum transparency required for readers to understand how the proposal was built.

 : CONCEPTUAL NARRATIVE REVIEW

**Response:**

 I have clarified in several parts of the study that this is a ‘conceptual narrative review’ because it primarily advocates for the adoption of a specific conceptual pedagogical design. This is done in the abstract section (page 2), and in the third paragraph of page 3 (introduction section). The revisions in the latter:

 “Drawing on relevant literature on experiential learning and faculty-led study abroad programs, this conceptual narrative review argues that short-term study abroad programs are relevant as a means to supplement what is taught in SARD classes. Considering the complex nature of the topic, which comprises dynamic tensions among Southeast Asian states due to haze pollution, the difficulty of balancing economic performances and environmental protection, as well as challenges of climate change, this study perceives that students could benefit from a short-term abroad program that helps them work on a specific case related to sustainability. As argued by many scholars in the past, experiential learning provides a unique learning atmosphere for students as they delve into the center of the community to observe by themselves, dynamics related to the subjects being taught in classrooms ( Below et al., 2021; Blewitt et al., 2021; Kolb, 1984; Pipitone, 2018; Strange & Gibson, 2017). As a means to bridge the conception of experiential learning in the SARD courses, this study is informed by Amy Below, Amanda Nydegger, and Mary Jane Parmentier’s ‘Experiential Learning through Faculty-led Study Abroad Programs’ and looks to implement the program conceptions of the study into SARD courses, by reviewing how experiential learning can respond to the unique challenges encountered in the teaching of SARD and sustainability.”

 Comment 2:

 My second critical concern involves the proportionality between evidence and conclusions. The manuscript plausibly argues that short-term study abroad programs could enrich sustainability learning in SARD courses. However, in the conclusion and in several parts of the discussion, the language at times approaches a claim of demonstrated feasibility or effectiveness. Since the article does not implement a program, does not report intervention outcomes, does not compare cohorts, and does not provide original empirical evidence on student learning, that degree of closure goes beyond what the design can actually support. I do not see this as a fatal flaw in the idea itself, but rather as a problem of claim calibration. To make the article scientifically stronger, the conclusions should be reformulated as a conceptually grounded and plausible proposal rather than as an already validated instructional platform.

**Response:**

 I have revised the conclusion section to accommodate the concern of Reviewer 1. In the second and third paragraphs of the conclusion (pages 12 & 13), I clarify that what is being narrated within the article is a conceptually grounded piece and they are in the form of a pedagogical recommendation (rather than being adopted and implemented in the past). The revisions:

 “This conceptual narrative review identifies the issue of teaching and learning sustainability topics within SARD courses as its main problem. Teaching sustainability presents distinct challenges, as students need to witness firsthand the issues undermining sustainability practices across the region. However, within existing teaching and learning systems, classroom teaching cannot offer direct experiences of the world regarding sustainability issues because it focuses on empirical and theoretical explanations. Therefore, drawing on relevant literature on experiential learning and faculty-led short-term study abroad programs, this study bridges the concept of experiential learning, as proposed by Below, Nydegger, and Parmentier, and its potential for adoption in the teaching and learning process of SARD courses. As scholars have argued in the past, experiential learning provides students with a unique learning environment, allowing them to observe firsthand the sustainability issues faced by communities and discuss potential solutions with their peers.

 Through the pedagogical proposal offered in this study, faculty-led study abroad programs, as mechanisms of experiential learning, potentially provide a feasible learning platform for students, considering several elements that the literature argues are important. Regarding the program’s outline, this study proposes several ideas for intertwining sustainability and Southeast Asia as the focus of the study abroad program. Although sustainability is a minor topic in an SARD course, the program offers the option to select several emerging sustainability challenges in Southeast Asia, which helps ignite students’ curiosity and understanding of the region. These include issues such as haze pollution, IUUF, and climate change, which are unique to the Southeast Asian context. As the literature also shows, it is essential to incorporate topics related to cultural awareness within the substantive content, considering that the study abroad program will take place in a country with which students may not be familiar.”

 Comment 3:

 My third observation concerns the comprehensiveness of the review. The manuscript includes recent and relevant references and demonstrates familiarity with current debates on sustainability education, service-learning, and study abroad. However, the literature discussion is not fully exhaustive and is not analytically systematized to a sufficient degree. In particular, the transition from barriers to sustainability learning, to alternative pedagogies, to the adoption of the Below, Nydegger, and Parmentier framework depends more on a reasonable argumentative bridge than on an explicitly traceable synthesis. It would be very helpful to include a correspondence table or conceptual diagram showing which strands of the literature support each component of the proposal: topic selection, pre-departure orientation, instructor responsibilities, student responsibilities, and possible pedagogical strategies during the program. This would substantially strengthen the manuscript’s internal coherence.

**Response:**

 I agree that the fourth section would benefit from a clearer sequence that better represents which strands of the literature support each component of the proposal. However, as shown in section 4, they are divided into three main sub-themes, each discussing different discourses for its sub-section. In the revisions, I clarify this in the first paragraph of section 4 (page 8) to show readers that there is relevant literature to consider before bridging the ideas of experiential learning. The revised paragraph:

 “The ideas introduced by Below, Nydegger, and Parmentier will be integrated into the teaching and learning process of SARD courses within IR programs. In doing so, the following will be structured as follows. First, it provides a recommendation for a program’s outline, aiming to intertwine the fundamentals of sustainability with Southeast Asian contexts for an abroad program. Following this is an elaboration of the instructors’ responsibilities and what is expected from a participating student. By providing such guidelines, this study benefits instructors who seek to adopt experiential learning and integrate creative pedagogies with service-learning in their teaching and learning practices. In each subsection, the section explores relevant literature that helps build the case for the potential success of adopting the pedagogical proposals related to sustainability learning. ”

 Comment 4:

 My fourth observation concerns the delimitation of the context of applicability. The article proposes this model as a useful alternative for SARD courses in Asian universities and even suggests that intra-regional mobility may reduce costs. However, the proposal relies on strong institutional assumptions: active international partnerships, instructor capacity for cross-border coordination, access to funding or realistic fundraising opportunities, and adequate logistical support. The text mentions some of these factors, but it does not clearly delimit that the viability of the proposal depends on such enabling conditions. In my view, this does not require redesigning the paper, but it does require making the implementation assumptions explicit so that readers do not interpret the proposal as universally applicable.

**Response:**

 I address Reviewer 1’s concerns in the first paragraph of page 10. It shows that the strong institutional assumptions (regarding partnerships and feasibility) are strongly determinant of how soon the instructor can confirm those points. Therefore, I revised the narrative to show that these are enabling conditions, and instructors need to consider this strongly. The revised paragraph:

 “As the responsible individual for the program, the instructor would then need to identify potential partner universities to conduct the short-term study abroad program. Instructors should acknowledge that their universities have established partnerships with other higher education institutions in the past, with some of these partnerships remaining active. Therefore, instructors should be able to identify host university options, taking into account that a particular higher education institution may have expertise and specialize in one of the themes the instructors wish to emphasize in the study abroad program. Nevertheless, an instructor should be able to weigh various considerations when determining the host country and university. For example, in a SARD course, instructors should first consider Southeast Asian Universities as the primary option if it is technically possible to make that long journey. As SARD courses are mainly offered in Asian higher education institutions, the bearing costs are not expected to be excessive. Instructors should address this point before the idea is introduced, as it will determine the proposal's viability depending on the enabling conditions.”

 Comment 5:

 My fifth important observation relates to the ethical and relational framing of the proposal. The manuscript states that no ethics statement is needed, which is understandable if the article is read strictly as a non-empirical piece without data collection. However, because the paper recommends forms of learning that may involve engagement with communities, local actors, and culturally sensitive environments, it would be valuable to distinguish between research ethics and ethics of pedagogical implementation. A brief expansion on this point would strengthen the manuscript, particularly regarding cultural preparation, relationships with host communities, and the prevention of extractive or instrumentalizing approaches to the local context.

**Response:**

 I agree with this statement. Therefore, I revised the last paragraph on page 10 to discuss that, in certain circumstances, ethical clearance may be required by the host university. This is especially true if students aim to conduct research or a project on sensitive topics. The revised paragraph:

 “If instructors are looking to adopt a more interdisciplinary approach to their studies, perhaps examining innovative learning systems could provide the solution they need. As Below’s study argued, “[…] interdisciplinary approaches can facilitate rich conversations and learning experiences and attract students” ( Below, Nydegger and Parmentier, 2021, p. 83). Therefore, it would be beneficial to refer to past studies on alternative methods in teaching and learning sustainability, such as through problem-based learning, community-based learning, or quizzes ( Below et al., 2021; Bremner & Steed, 2025; Carrió Llach & Llerena Bastida, 2023; Howell, 2021; Nguyen et al., 2024; Shah et al., 2024). Meanwhile, instructors could also explore more established teaching and learning methods that are commonly used in experiential learning, such as service learning. As the literature indicates, service learning is a pedagogy that can enhance students’ awareness of a specific topic ( Ribeiro et al., 2023; Álvarez-Vanegas, Ramani, and Volante, 2024; Aramburuzabala, 2025). In the context of SARD, students can gain an understanding of sustainability by exploring the community through specific projects that expose them to real-life sustainability phenomena in the Southeast Asian context. Instructors should also be aware of the potential need of ethical clearance from their host university, if it is needed in the process of researching or conducting projects on sensitive topics. ”
